# Spectral light quality regulates photosynthesis and thylakoidal protein complexes to improve drought tolerance in okra rootstocks

**DOI:** 10.3389/fpls.2025.1706708

**Published:** 2026-01-05

**Authors:** Preethika Suresh, Srinivasan Rameshkumar, Kyung Hee Lee, Dong Won Bae, Sowbiya Muneer

**Affiliations:** 1 Horticulture and Molecular Physiology lab, Department of Horticulture and Food Science, School of Agricultural Innovations and Advanced Learning, Vellore Institute of Technology, Vellore, Tamil-Nadu, India; 2 School of Bioscience and Technology, Vellore Institute of Technology, Vellore, Tamil Nadu, India; 3 Department of Horticulture, Annamalai University, Chidambaram, Tamil-Nadu, India; 4 Central Instrument Facility, Gyeongsang National University, Jinju, Republic of Korea

**Keywords:** drought stress, light spectra (R:B), physiological traits, cellular processes, stress tolerance

## Abstract

**Introduction:**

Global agriculture is seriously threatened by drought stress, especially in arid and semi-arid areas where crop production is already low. Okra (*Abelmoschus esculentus* L.), a nutritionally beneficial yet underutilized crop, experiences considerable productivity and quality losses. Plant cellular activities are modulated by light quality, which impacts stress adaptation. To increase okra productivity and stress resilience, this study attempts to optimize the performance of drought-resilient rootstocks under various spectral light conditions.

**Methods:**

Two okra genotypes, NS 7772 and NS 7774, were evaluated in this study under drought conditions with two light spectra: red: blue: white (R: B) light-emitting diodes (LEDs) and white light (WL).

**Results and discussion:**

Infrared thermographic images showed lower canopy temperatures, indicating increased water content under R:B light conditions. NS-7774 exhibited improved drought tolerance confirmed by decreased malondialdehyde (MDA) levels and enhanced antioxidant enzyme activity. Metabolic stability was indicated by preserved photosynthetic protein complexes and stress-responsive polypeptides in the thylakoidal multiprotein complex profiling. Nutrient preservation was validated through SEM-EDAX analysis, assessments of chlorophyll, total soluble protein, isoenzyme patterns, and antioxidant activity (CAT, SOD, APX, GPOX). Multivariate analysis highlighted six critical factors contributing to resilience. These results demonstrate that combinational spectral light modifications can greatly increase the tolerance of okra to drought. The superior performance of NS 7774 under R:B light conditions suggests the potential suitability of NS 7774 for cultivation in drought-prone zones of India.

## 1 Introduction

The National Centre for Environmental Information’s annual report states that, in 2024, 2.32 degrees Fahrenheit was the average surface temperature worldwide, leading to increased abiotic stressors for crops (World Meteorological Organization, 2025). Indeed, these are the primary causes of crop failure globally, reducing average yields for the majority of important crops by over 51%–82% (Kopecka et al., 2023). Around 9% of the world’s land is affected by drought (Minhas et al., 2017). Drought events have become more frequent and intense in recent years due to the ongoing increase in global temperatures (Zhou et al., 2024). Okra (*Abelmoschus esculentus*) is a nutrient-dense vegetable that contains dietary fiber, mucilage, essential amino acids (like lysine and tryptophan), and linolic acid. It contains essential vitamins (A, B-complex, and C) and minerals (calcium, magnesium, iron, and potassium). Okra, which is high in polyphenols and flavonoids, has strong anti-cancerous, antidiabetic, and antioxidative properties (Elkhalifa et al., 2021). India tops the table in okra production with more than seven million tons and one thousand (Int$) gross value in 2023 (FAOSTAT, 2025). With almost two-thirds of its land susceptible to drought, India is extremely vulnerable. However, Kenya tops in export quantity (>15,000t) and quality, with export value of up to 44,039 in 2023 (FAOSTAT, 2025). The yield is decreased when bhendi is grown during hot and dry seasons because it experiences flower drop and premature fruit drop (Elkhalifa et al., 2021). Therefore, in order to create better and climate-resilient crops and implement strategies to address changing climatic circumstances, a mechanistic understanding of abiotic stress in global agriculture is essential (Minhas et al., 2017).

Grafting modulates a plant’s physiological and molecular/cellular strategy, can activate *DREB, NAC*, and *WRKY*, which regulate protective mechanisms including antioxidant enzyme activity and osmolyte accumulation (Erpen et al., 2018; Yu et al., 2021). Under water deficit conditions, grafted plants’ increase the production of ABA, proline, soluble sugars, and polyamines aids in osmotic adjustment, ROS detoxification, and better stomatal regulation (Erpen et al., 2018; Markus et al., 2025). Additionally, effective hydraulic conductivity and hormonal signaling are supported by the rootstock–scion interaction (dos Santos Severino, 2015; Wang et al., 2021). Finding the right rootstock is essential to increasing graft success during drought (Zhao et al., 2022; Bondok et al., 2022). Hence, grafting, underpinned by molecular-level rootstock selection, is an eco-efficient strategy to enhance drought resilience in crops like okra. However, Graft incompatibility, disease susceptibility, environmental sensitivity, and the requirement for expert labor are some of the major obstacles in grafting. Successful graft establishment and crop performance are further hampered by inadequate vascular connection, hormonal imbalance, and restricted access to training and ideal healing circumstances (Suresh and Muneer, 2025).

Light-emitting diodes (LEDs), such as Blue, red, and far-red wavelengths, influence important genes, including *CRY, PHOT, PHYA, PHYB*, and *PIF*s, which in turn promote callus development, cell division, and vascular differentiation (Khan et al., 2025: Sharma et al., 2021). LED light promotes auxin-mediated cell differentiation and photosynthesis, whereas blue light boosts photomorphogenesis and secondary metabolite synthesis (Han et al., 2025; Shahzad and Gul, 2025; Xu et al., 2018). Transcription factors, electron transport proteins, and genes encoding chloroplast components have been found to be important targets for improving photosynthetic efficiency and stress tolerance in vegetables (Theeuwen et al., 2022). Additionally, LED lighting technology’s environmental optimization offers more chances to optimize plant performance. In particular, it has been demonstrated that red LED light at 660 nm and blue LED light at 435–450 nm improve stomatal conductance, chlorophyll synthesis, and total biomass accumulation in lettuce and other vegetable crops (Alrajhi et al., 2023). In order to coordinate the physiological processes essential for the development and healing of graft unions, this study uses light-driven signals interacting with hormones such as auxin, cytokinin, gibberellin, ethylene, and jasmonic acid to improve grafting efficiency and healing. Through phytochrome signaling, red light performs a major role in drought adaptation. Phytochromes regulate PIN-mediated auxin transport to reinforce root extension while concurrently increasing NCED-dependent ABA production and encouraging more decisive stomatal closure (Ren et al., 2023). Plants can access reduced soil moisture reserves and maintain physiological activity during extended stress by using deeper rooting as an avoidance strategy when exposed to red light. Far-red radiation influences phytochrome photo equilibrium and increases root: shoot ratios, improving carbon allocation toward root biomass, a trait associated with higher water uptake efficiency (Gul et al., 2025; Chen et al., 2024). These interactions suggest that white light provides crucial background spectral information that sustains circadian and photosynthetic homeostasis during stress.

The current research aids in optimizing various light spectrum potentials in identifying drought-resilient rootstocks in okra. The study investigates the effect of spectral quality (red:blue: white R:B vs. full-spectrum white light-WL) on photosynthetic performance in two okra genotypes, NS 7772 and NS 7774. By integrating physiological, biochemical, proteomic, and image analyses, the research aims to optimize spectral light-driven graft healing and identify drought-resilient rootstocks. The outcomes of this work offer valuable insights for enhancing okra cultivation in drought-prone environments through grafting and light spectrum management.

## 2 Materials and methods

### 2.1 Plant material, spectral composition, and experimental design

**Figure 1:**
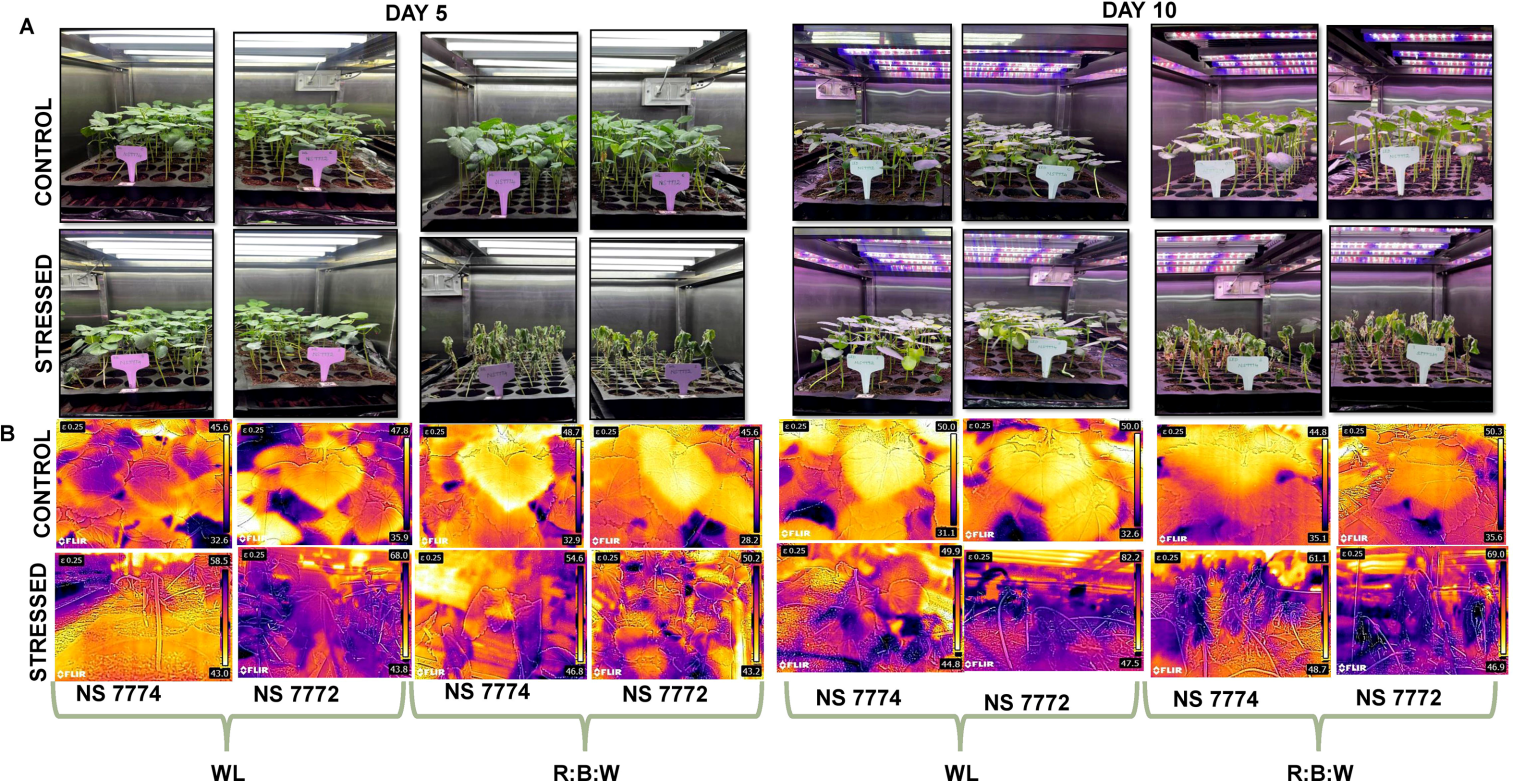
**(A)** Completely randomized experimental design setup at white fluorescent light system and multi-spectrum LED light system at controlled climatic conditions with Relative humidity of 65% and temperature of 25 degrees **(B)** Infrared thermographic images depicting elevated canopy temperature distribution indicate reduced transpiration and relative water content during day 5 and day 10 of drought stress.

The experiment was conducted in an automated walk-in growth chamber (LABTOP: LGC-60WP). The cycle was maintained at 25°C and 70% humidity in the plant growth chamber. The Photon flux densities with wavelengths of 380–730 nm and 360–780 nm were 55–58 mol/cm/s and 75–77 μmol/cm/s, respectively, for red: blue: white (R: B) and white light (WL) lighting systems. For R: B planting, the light intensity was set to 3000 ± 50 lux, and the LED light ratio was White: Red: Blue 5:4:1. The plants for morphological observations were grown in trays, and for biochemical assessments, individual pots per plant were used. Red soil, sand, and vermi-compost were combined in a 1:1:1 ratio to create the potting mixture. Each of the protray cells (10.9*10.9 cm) holds 400 g of pot mix (**Figure 1a**).

The research study used a completely randomized design (CRD) with two distinct light spectral combinations: 100% white fluorescent light (WL) and 4:1:5 red, blue, and white (R: B) light. Two distinct okra genotypes (purchased from certified government seed vendor, Namdhari Seeds Pvt. Limited, Vellore), viz, NS7774 and NS7772, were sown (seed rate: one per portray cell) under each lighting system and divided into two groups: stressed (treated with drought) and control (scheduled irrigation). At the vegetative stage (20 days after sowing), drought treatment was given by withholding of water, and leaf samples were collected after day 5 and day 10 of stress and immediately frozen in liquid N_2_ and stored in a deep freezer at -80°C for further biochemical and proteomic experimental analysis. Two sets of 240 pots were created from a group of 480 pots that had been filled for germination (pH-5.2-6.3; EC- 0.8–2 Ds/m; refer to**Supplementary Figure 2**. Each set was exposed to separate R: B and WL light with 16 hours of photoperiod and 8 hours of darkness. A total of 120 protrays cells with four replicates were assigned control and stressed under R: B and WL light spectrum for morphological and physiological experiments. For all the experiments, analytical and molecular-grade chemicals were used with three biological replicates for proteomic studies and four biological replicates for biochemical experiments. For overall study control plants (NS 7774 and NS 7772 grown under both light spectrums) represents positive control. Whereas, control plants (NS 7774 and NS 7772) grown under only white light (WL) represents negative control. The severity of drought stress conditions were observed based on the weight of trays and morphological observations (dried leaves) and also confirmed by raise in EC levels (**Supplementary Figure 2)**.

### 2.2 Morphological and photosynthetic observations

For ten days in consecutive days, all of the potted crops were treated under regulated and drought-stressed conditions, and their morphology was regularly observed and photographed using FLIR-TG165-X (Thermal Imaging Inspection Camera) (**Figure 1**). The individual plant morphology is represented in **Supplementary Figure 2**. Using established procedures, the morphological parameters such as fresh weight, dry weight, root and shoot length were meticulously measured. The plants and their roots were removed from the pots, thoroughly cleaned with distilled water, and then weighed on a computerized weighing balance to ensure precise measurements for the fresh weight. Using a ruler, the length of the root and the shoot standard was determined, and the results were documented. The stem girth was estimated using a vernier calliper. Photosynthetic parameters, such as net photosynthetic rate, stomatal conductance, and transpiration rate, were measured using the portable SPAD (Konica Minolta, Tokyo, Japan).

Using Heath and Packer (1968) thiobarbituric acid reactive substances (TBARS) assay, the amount of malondialdehyde (MDA) was determined. In a mortar and pestle that had been chilled, 500 mg of fresh leaf material and 5 ml of 0.1% tri-chloroacetic acid (TCA) were pulverized together. For ten minutes, the homogenate was centrifuged at 329 xg. A separate test tube was filled with 4 mL of 0.5% TBA and 1 mL of supernatant, then incubated for 30 minutes at 95°C in a boiling water bath. A UV spectrophotometer was used to measure absorbance at 532 nm and 600 nm after the reaction was swiftly stopped on ice. Proline estimation was carried out by homogenizing 0.3 g of fresh leaf samples with 3 mL of 3% aqueous sulfosalicylic acid. The tubes with the homogenized materials were centrifuged for 10 minutes at 4°C at 117 xg using a NEYA 16 R REMI. Following centrifugation, two milliliters of acid ninhydrin solution (which contained ortho-phosphoric acid and glacial acetic acid) and two milliliters of glacial acetic acid were added to around two milliliters of supernatant. The mixture was then incubated at 95°C in a boiling water bath. To stop the further reaction, the test tubes were submerged in an ice bath after 60 minutes. Each test tube was filled with 4 milliliters of toluene after it had been allowed to cool to room temperature. Using a UV spectrophotometer and toluene as a blank, the absorbance of each mixture was measured at 520 nm (Razi and Muneer, 2023). The MDA content was calculated using Equation 1.

(1) $$\begin{array}{c} MDA\;content(\frac{nmol}{gm}FW)=(A532-A600)*volume*\frac{1000}{155}*fresh\;wt \end{array}$$

### 2.3 Water status and transport

Barrs and Weatherley (1962) is used to calculate the Relative Water Content (RWC). The leaf samples’ fresh weights were recorded as fresh weight (FW) as soon as they were cleaned with distilled water. These samples were then immersed in distilled water for two hours, and their weight was recorded as turgid weight. Following a 24-hour drying period at 65°C in a hot air oven, the leaf samples’ dry weight (DW) was determined. The relative water content % (RWC) was calculated using the Equation 2,

(2) $$\begin{array}{c} Relative\;water\;content\;(\%)=\frac{Fresh\;wt.-Dry\;wt.}{Turgid\;wt.-Dry\;wt.}*100 \end{array}$$

Samples of fresh plants were pulled and thoroughly cleaned. After being fixed for five to six hours in a 0.1% safranin, they were washed with water. Using a sharp surgical razor blade, the stem of each genotype was divided into transverse sections, which were then examined using a dark field and phase contrast microscope (model: MT4300L, MEIJI TECHNO CO., LTD., Kyoto, Japan).

### 2.4 Antioxidant enzyme assay and their native PAGE profiling

The antioxidant enzyme assays were followed according to Venkat et al. (2023) with slight modifications. For ascorbate peroxidase (APX) (EC 1.11.1.11) and catalase (CAT) (EC 1.11.1.6) enzyme extraction, a 50 mM sodium phosphate buffer with 1 mM EDTA was prepared after combining 0.05% Triton X100 and 2% polyvinylpyrrolidone (PVP). For APX activity, 0.2 g of the leaf sample was homogenized using 2 ml of extraction buffer. The ground samples were centrifuged for 25 minutes at 4°C and 1624 xg. Next, 600 μl of reaction buffer containing 0.1M KH_2_PO_4_ and K_2_HPO_4_, 100 μl EDTA, 100 μl ascorbate, and 100 μl 3 mM H_2_0_2_ to 0.1 ml of supernatant. By measuring the absorbance at 290 nm for three minutes at 30-second intervals, the drop in ascorbate content was quantified (E = 2.8 mM^−1^ cm^−1^). For visualizing the isoenzyme patterns of each antioxidant enzyme, native PAGE profiling was carried out for APX, CAT, sodium oxide dismutase (SOD) (EC 1.15.1.1) and glutathione peroxidase (GPOX) (EC 1.11.1.9) following Razi and Muneer (2023), with slight modifications. For APX, 200 mg of fresh leaf material was homogenized with 2ml of extraction buffer (pH 7.8) containing 50mM potassium phosphate buffer, 1mM EDTA, 1mM phenylmethylsulphonyl fluoride (PMSF), 5mM ascorbate and 2% polyvinylpyrrolidone (PVP). Later, it was centrifuged at 3654 xg for 25 mins. The homogenized drought-stressed and control samples with replicates were considered for the study, each taken in a 3:1 ratio (sample: dye) for APX and CAT. The dye includes 240 mM of Tris-HCl, 40% of glycerol and 5% of bromophenol blue in 10% and 4% of resolving/separating and stacking buffer, respectively. For around three to four hours, the electrode buffer (pH 8.3) with 25 mM Tris-base and 192 mM glycine was kept at 4°C and 80 V. The gel was equilibrated with 50 mM phosphate buffer (pH 7) and 20 mM ascorbate for 30 minutes. Later, incubated with a solution containing 50mM phosphate buffer, 4mM ascorbate and freshly prepared 2mM H_2_O_2_ for 20 mins. Later washed with phosphate buffer for a minute (pH 7) and later stained with a solution containing 50 mM phosphate buffer, 1 mM of EDTA, 33.2 µm of riboflavin, 0.2% of TEMED, and 0.245 mM nitro blue tetrazolium chloride (NBT) for 15 minutes. After that, the gel was exposed to light in distilled water for roughly five minutes. The distilled water was thrown away as soon as the bands appeared, and the gel was submerged in 6% acetic acid to stop the reaction. A trans-illuminator was used to further visualize the bands with isoforms.

The catalase (CAT) enzyme activity was measured by homogenizing 0.2 g of leaf material with 2 ml of 0.5 M extraction buffer (pH 7.5), which contained 1% PVP, Triton-X100, K_2_HPO_4_ (dibasic potassium phosphate), KH_2_PO_4_ (potassium dihydrogen phosphate), and 1 mM EDTA (ethylene diamine tetra acetic acid). The ground samples were centrifuged for 20 minutes at 4°C and 2745 xg. The supernatant was then extracted independently. Around 600 μl of 0.05 M reaction buffer containing phosphate buffer with pH 7.3 was added along with 200 μl of enzyme extracts. Ultimately, a total volume of 1 ml was achieved by manually mixing 3 mM of H_2_O_2_ while measuring absorbance. At 30-second intervals, the absorbance was measured for three minutes at 240 nm (E = 39.4 mM^− 1^ cm^−1^). The activity of the superoxide dismutase (SOD) enzyme, a 0.2 g sample of fresh leaves was taken and homogenized using a pre-cooled mortar and pestle with 2 ml of 0.5M extraction buffer containing phosphate buffer (K_2_HPO_4_ (dibasic potassium phosphate), KH_2_PO_4_ (potassium dihydrogen phosphate) (pH 7.5), 1% of PVP (polyvinyl pyrrolidone), 1% of Triton-X 100, and 1 mM EDTA. The supernatant was obtained by centrifuging the ground materials for 15 minutes at 3654 xg and 4°C. The supernatant was combined with 1.5 ml of reaction buffer that contained distilled water, 200 mM methionine solution, 2.25 mM NBT solution, 3 mM EDTA, 60 μM riboflavin, 0.1 M sodium carbonate, and 0.1 M phosphate buffer (pH 7.8). One set of this mixture was incubated at ambient temperature in a dark environment, and the other set was incubated for ten minutes under the light of a 15 W fluorescent lam Except for the enzyme extract, a blank was kept in both situations. At 560 nm, the sample’s absorbance was measured. A unit of enzyme activity, measured in enzyme mg−1 protein h−1, is equivalent to the percentage decrease in color. The extraction buffer containing 100mM phosphate buffer (pH 7.8), 1mM EDTA, 3mM dithiothreitol, 5% PVP was homogenized with 500mg of tissue. Later, were centrifuged at 1790 xg for 30 mins. The sample to dye ratio was 2:1. The same sample was used for staining SOD and CAT isoenzyme Patterns. For SOD, a native gel of 15% resolving and 4% stacking gel was prepared. After the gel ran for 3-4hrs at 70V, the gel was stained with 50mM phosphate buffer, 1mM EDTA, 33.2 μm riboflavin, 0.2% TEMED, 0.245 μm NBT solution for 30 min. For CAT, native gel of 10% resolving and 4% stacking was prepared, followed by immersing the gel in 3.27 mM H_2_O_2_ for 25 mins. Later, the gels were washed with distilled water for 2–3 seconds and again stained with 1% Potassium ferrocyanide (K_3_Fe(CN)_6_) and Ferric chloride solution. The SOD native gel was irradiated in light after staining for 5 mins in distilled water and then visualized in UV transilluminator and preserved in 6% acetic acid solution. For GPOX staining, native gel of 12% resolving and 4% stacking was prepared. After the gel runs, the gel was soaked in 50mM Tris-HCl buffer (pH 6.8) for 10 mins followed by staining in 50mM Tris-HCl buffer (pH 6.8), 0.46% (v/v) guaiacol and 13mM H_2_O_2_. Once the bands were visible prominently, the photograph was documented.

### 2.5 H_2_O_2_ and O_2_^^-^^*In-situ* localization

All of the fresh leaf samples were placed in petri plates containing a 1% solution of 3,3-diaminobenzidine (DAB) in Tris-HCl buffer (pH 6.5) to detect H_2_O_2_ localization. This was followed by a 5-minute vacuum infiltration and a 16-hour dark incubation period at room temperature. In order to characterize the reaction of DAB (3,3-diaminobenzidine) with H_2_O_2_, leaves were submerged in ethanol for the bleaching process and then maintained in a hot water bath at 65°C until brown stains developed. Fresh leaves were obtained and submerged on petri plates filled with a 0.1% solution of nitro blue tetrazolium (NBT) in K-phosphate buffer (pH 6.4) containing 10 mM Na-azide to detect O_2_.^^-^^ After five minutes of vacuum infiltration, illumination was continued until the dark blue spots, which are indicative of blue formazan precipitate, appeared. The leaf samples were bleached in boiling ethanol and then photographed as previously mentioned.

### 2.6 Stomatal structure analysis using SEX-EDAX

The leaves’ thin outermost layer was carefully removed and put on the glass slide. A cover slip was placed over a few drops of food coloring agent and distilled water for examination at 10x and 40x magnifications using a phase contrast microscope (model: MT4300L, MEIJI TECHNO CO., LTD., Kyoto, Japan). On the fifth and tenth days of treatment, fresh plant samples were collected, and the fresh leaves were then sliced to a <1 cm square so that the stomatal structure could be examined. The leaves were then fixed for two to three hours at a pH of 7.4 in the first fixative solution, glutaraldehyde. The samples were dehydrated using an ethanol series ranging from 95% ethanol to 50% ethanol as the next step, once the fixation was completed. The following step involved utilizing a scanning electron microscope (model: EVO-18 Research, Carl Zeiss, United States of America) to visualize the stomata’s structure. Energy dispersion X-Ray Spectroscopy was carried out to understand the stomata’s and the surrounding cells’ elemental makeu This integration proves beneficial in the investigation of stomatal functionality, responses to environmental alterations, and the effects of drought treatments on plant morphology.

### 2.7 Proteomic profiling using BN-PAGE

The phosphate buffer was used to homogenize 500 mg of fresh leaf samples. Centrifugation was carried out at 1624 × g rcf for 20 minutes at 4°C following enzyme extraction, and the resulting supernatant was collected for protein and enzyme tests. The Bradford test was used to obtain the standard curve, and the enzyme aliquot’s protein content was ascertained.

With a few minor adjustments in accordance with our earlier methodology (Razi and Muneer, 2021), the analysis of native protein in thylakoids by 1D BN-PAGE was carried out in accordance with the prior study (Razi et al., 2021). After about 4 g of leaf sample was ground up in liquid nitrogen, it was homogenized with 4 ml of B1 buffer (pH 7.8) that had been refrigerated beforehand and contained 20 mM tricine, 0.3 mM sucrose, and 5 mM MgCl_2_. After that, a mira cloth was used to filter the mixture. Centrifugation was carried out for 10 minutes at 4°C and 329 xg. Following centrifugation, the pellet was put in 4 milliliters of B1 buffer and centrifuged for 10 minutes at 329 xg at 4°C. The pellet was centrifuged at 329 xg for 10 minutes at 4°C after being re-suspended in 4 ml of B2 buffer (pH 7.8) that contained 20 mM tricine, 70 mM sucrose, and 5 mM MgCl_2_. The thylakoid membrane is found in the third pellet. After centrifuging the third pellet for two minutes at 4°C at 199 xg, wash it twice with 3 ml of washing solution that contains 330 mM sorbitol and 50 mM bis-tris. Following washing, the final pellet was carefully dissolved in 2% w/v n-dodecyl-ß-D-maltoside to solubilize it. It was then combined with 0.1% loading dye (5% CBB-G250, 100 mM Bis-Tris-HCl, pH 7.0, 30% w/v sucrose, and 500 mM ϵ-amino-n-caproic acid). The protein content was measured using the Bradford assay. The protein samples obtained from the aforementioned procedures were put onto a 1.5 mm 7.5–12% w/v acrylamide gradient gel. Using a Protean II xi cell electrophoresis system (Bio-Rad, Hercules, CA, USA) and a constant voltage between 80 and 100 V for 4–6 hours until the gel run was finished, 1D BN-PAGE was carried out by running the gel electrophoresis at 4°C.

### 2.8 Statistical analysis

The statistical analysis was performed using statistical analysis software (JMP Pro 18). Four biological replicates were used in a fully randomized manner, and the results were reported as the mean ± SE. A two-way analysis of variance and a Student’s t-test were used to assess differences across all treatments, with p < 0.05 serving as the significance level. According to Wirojsirasak et al. (2024), Linear connections between physiological and biochemical variables were measured using Karl Pearson’s correlation, with significance tested at p < 0.05. To find the main components causing trait variability and treatment clustering, principal component analysis (PCA) was performed across 19 variables (Patel et al., 2023).

## 3 Results

### 3.1 Infrared thermographic alterations in canopy temperature

Okra genotypes NS7774 and NS7772 showed distinct morphological responses to combined red:blue: white LED (R: B) and white light (WL) treatments under controlled light system (**Figure 1**). In contrast to R: B, which produced shorter stature, thicker stem girth, and dark green foliage, WL caused plants to be taller with thin stems and light green leaves. Additionally, R:B improved root architecture and diameter, indicating better resource collection. Early fruiting occurred within 20 days under R: B, compared to 27 days under WL. Notably, stress deposition was indicated by the observation of white amorphous spherical nano-sized particle deposition underneath the leaf structures, specifically under WL following drought stress (**Supplementary Figure 1**). This was possibly due to calcium oxalate crystal formation resulting from oxidative and photooxidative stress caused by the light spectrum, especially high-intensity white light (Minocha and Long, 2023). In order to chelate extra calcium ions and avoid cytotoxicity, plants store oxalate. Under abiotic stress, these crystals control cellular ion homeostasis and serve as a calcium reservoir. However, R: B light changed the morphology of plants, making them more drought-tolerant and structurally resilient. This was especially true for NS7774, which showed a better sensitivity to R: B spectral modulation. **Figure 1** represents the thermal infrared images of okra genotypes under different spectra. Under white light, the results indicate that NS7774 can serve as a drought-tolerant rootstock. Despite an increase in temperature to 50.75 on day 5 (compared to 39.1 in the control), it managed to reduce the temperature to 47.35. The drought sensitivity of NS7772, is showed by initial average leaf temperature of 36.9 increased to 55.9 on day 5 and 64.85 on day 10. The average leaf temperature of stressed NS7774 okra leaves under LED circumstances was 50.7 on the fifth day of stress, which is much higher than the control (40.8), but it dropped to 39.95 on the tenth day, suggesting that it may be a drought-tolerant rootstock. A similar pattern with more temperature volatility was seen in NS7772. On day five, the stressed leaf’s average temperature was 46.7, which was higher than the control’s (36.9). However, on day ten, it rose to 57.95, further demonstrating its susceptibility to drought. Therefore, NS7774 functions as a more effective drought-adapted rootstock when illuminated by R: B LEDs.

### 3.2 Morphometric trait assessment

**Figure 2:**
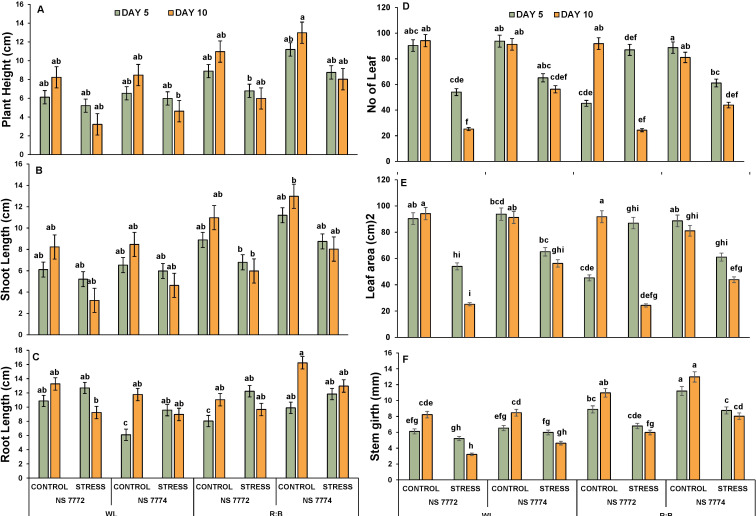
Plant architecture in response to drought **(A)** Plant Height **(B)** Shoot height **(C)** Root Length **(D)** No:of:Leaf, **(E)** Leaf area (cm)^2^ and **(F)** Stem girth (mm) of okra genotypes (viz., NS 7772 and NS 7774, along with respective controls) under white fluorescent light system (WL) and multi-spectrum LED (R:B) light system at controlled micro-climatic conditions. Vertical bars indicate mean ± SE for n = 4. Means denoted by different letter are significantly different at p ≤ 0.05 according to the Tukey’s studentized range test.

The effect of drought stress and spectrum light quality on plant architecture was evaluated using six crucial morphological traits: plant height, shoot height, root length, number of leaves, leaf area, and stem girth. On Days 5 and 10, drought stress significantly reduced the overall plant height in both genotypes under WL. However, plants cultivated under R: B LED light showed decreased suppression of height, especially in NS 7774, suggesting that growth inhibition is mitigated by light (**Supplementary Figure 3**). Among stressed plants, NS 7774 under R: B LEDs maintained the tallest plant on Day 10, indicating improved resistance to drought. The trend of shoot height was identical to that of overall plant height **Figure 2** In comparison to NS 7774, NS 7772 showed a larger decline under stress. In comparison to WL, the R: B light treatment promoted greater shoot elongation in both genotypes. By Day 10, NS 7774 had recovered significantly, indicating higher shoot growth plasticity. Under drought stress, root length was often more retained, especially in NS 7774 under R: B light. The longest roots were found on Day 10 in NS 7774 under LED-stressed treatment, which may have been an adaptive response for improved water foraging. Treatments resulting from drought stress showed a significant decrease in the number of leaves. However, R: B Led to had more leaves than WL, particularly in NS 7774. By Day 10, NS 7774’s leaf retention had significantly improved, indicating its ability to adapt morphologically and withstand spectrum-modulated stress. Leaf area substantially dropped under drought stress, especially in NS 7772 under WL. On Days 5 and 10, NS 7774 under R: B LEDs retained a larger leaf area than other stressed groups, indicating reduced leaf senescence and better turgor preservation. Stem girth was negatively impacted by drought, with NS 7772 under WL showing a notable decline. R: B light treatments, however, mitigated this decline in both genotypes. Under R: B LEDs, NS 7774 once more showed improved stem girth retention, especially on Day 10. However, NS 7774 outperformed NS 7772 in all morphological parameters under dry conditions, particularly under R: B LED lighting. R: B light had a favorable effect on growth characteristics, indicating a spectrum-induced regulation of morpho-physiological drought responses.

### 3.3 Relative water status and transport efficiency

**Figure 3:**
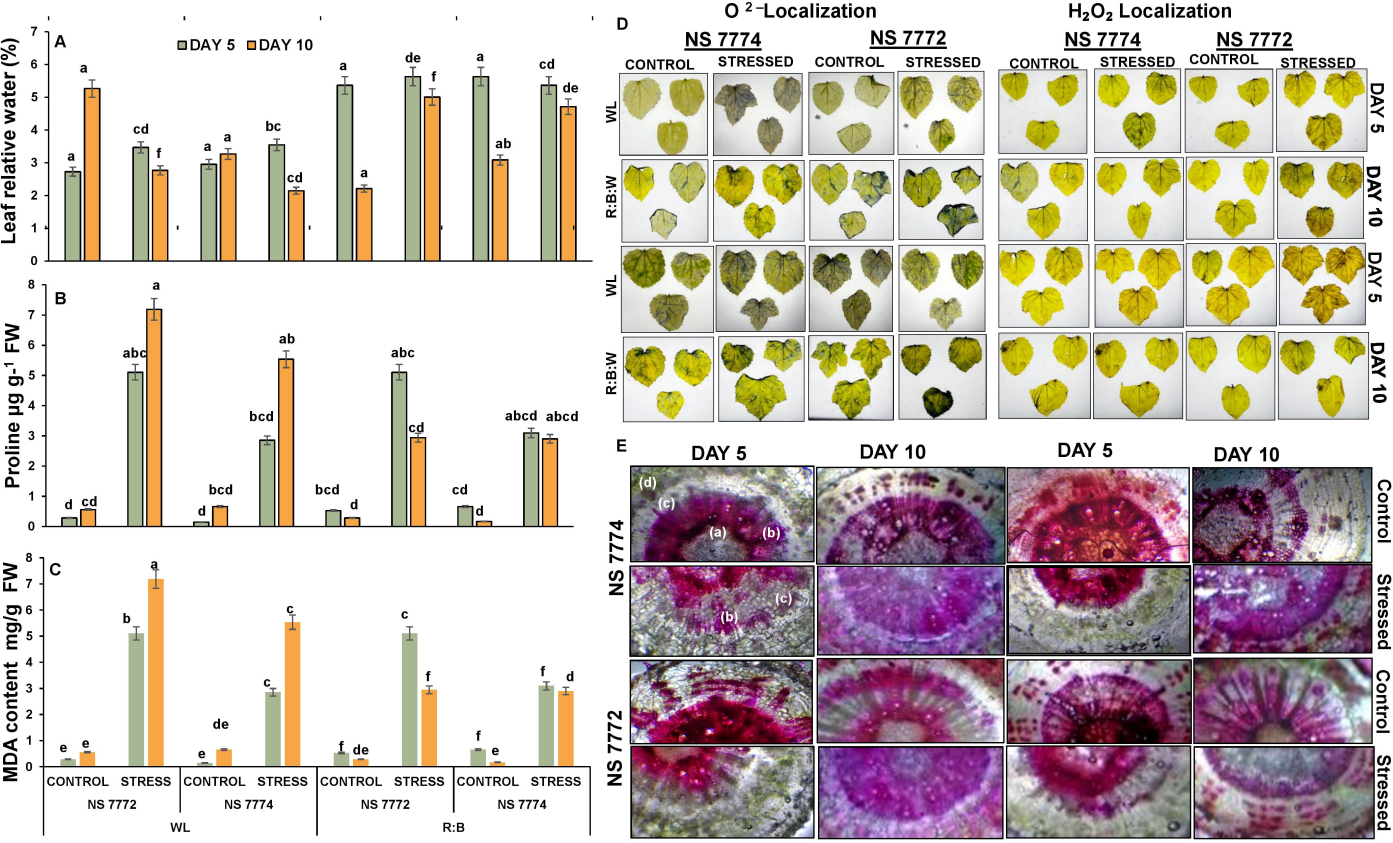
Physiological **(A)** Leaf relative water content, and oxidative stress responsors **(B)** Proline, **(C)** Malondialdehyde (MDA) content **(D)** Reactive oxygen species (ROS) differential accumulation (brownish spots - localization of H_2_O_2_ stress marker; bluish spots - localization of O2^−1^ marker) and **(E)** representing relative water transport by vascular bundles [a) Phloem; b) Xylem; c) Cortex; d) Epidermis respectively] of okra genotypes (viz., NS 7772 and NS 7774, along with respective controls) under white fluorescent light system (WL) and multi-spectrum LED (R: B) light system during drought stress. Vertical bars indicate mean ± SE for n = 4. Means denoted by the different letters are significantly different at p ≤ 0.05 according to the Tukey’s studentized range test.

Drought stress resulted in a considerable decrease in RWC in NS 7772 and NS 7774 when compared to their respective controls (**Figure 3A**). However, the extent of decrease differed between genotypes and light treatments. On Day 5, RWC declined significantly in NS 7772 under WL, indicating fast dehydration. NS 7774 under R: B LEDs had a considerably greater RWC than all other stressed groups, indicating improved water conservation and cellular turgor preservation. By Day 10, the RWC decline had accelerated in all groups; however, NS 7774 under LED light still had a higher RWC than its WL counterpart and NS 7772 under both lighting conditions. Anatomical cross-sections of okra stems were examined for vascular architecture and water transport integrity (**Figure 3E**). Both genotypes of control plants preserved intact xylem and phloem structure, as well as obvious vascular distinction, under both light treatments. Under drought stress, NS 7772 under WL exhibited collapsed xylem arteries, disordered cortex, and constricted phloem tissues, indicating poor water transfer and structural integrity. In contrast, under R: B LEDs, NS 7774 maintained well-structured and open xylem conduits, robust phloem strands, and compact cortical cells. This shows that vascular function and water conduction are improved even when stressed. On Day 10, NS 7774 under LEDs showed very little damage, suggesting tissue-level adaptation to preserve hydraulic conductivity, while NS 7772 under WL showed more severe structural deterioration.

### 3.4 Oxidative stress indicators

In response to drought, proline, a known osmoprotectant and oxidative stress mitigator, significantly increased in all treatments (**Figure 3B**). Due to drought, proline content increased slightly in both genotypes on Day 5, while NS 7774 under R:B LEDs had the highest levels. In comparison to NS 7772 under WL, the proline level of stressed plants in NS 7774 under LED light tripled by Day 10. While NS 7772, accumulated less proline over time and under both light conditions. This indicates that NS 7774 has more active proline biosynthesis stimulation, particularly when exposed to R: B light, which helps with stress reduction and osmotic adjustment. Both genotypes experienced a sharp rise in malondialdehyde (MDA) levels under drought stress, a sign of lipid peroxidation and membrane damage (**Figure 3C**). All stressed treatments showed a rise in MDA levels on Day 5, but NS 7772 under WL showed noticeably higher levels, suggesting early onset oxidative stress. Less membrane damage was indicated by NS 7774’s lowest MDA level under R: B LEDs. By Day 10, both light treatments had enhanced MDA accumulation in NS 7772, with WL causing the most severe peroxidative damage. NS 7774 exhibited much lower MDA levels under LED lighting, which correlated to better antioxidant protection.

By uptaking DAB and NBT stains, histochemical staining revealed varying quantities of superoxide (O_2_^-^) and hydrogen peroxide (H2O_2_) (**Figure 3D**). Drought-stressed treatments displayed bluish O_2_^-^ and brownish H_2_O_2_ spots on Day 5. The most notable ROS localization was seen in NS 7772 under WL, especially in mesophyll tissues. NS 7774, on the other hand, exhibited little ROS buildup under R: B LEDs, and fainter staining patterns suggested more efficient ROS scavenging mechanisms. All stressed treatments produced higher ROS levels by Day 10, but NS 7772 under WL exhibited severe oxidative damage, as evidenced by thick brown and blue deposits in the leaf and vascular regions. Interestingly, NS 7774 under LED lighting continued to exhibit reduced ROS levels even on Day 10, demonstrating continued redox equilibrium and antioxidant defense.

### 3.5 Antioxidant enzyme activity and isoenzyme profiling as a response mechanism

**Figure 4:**
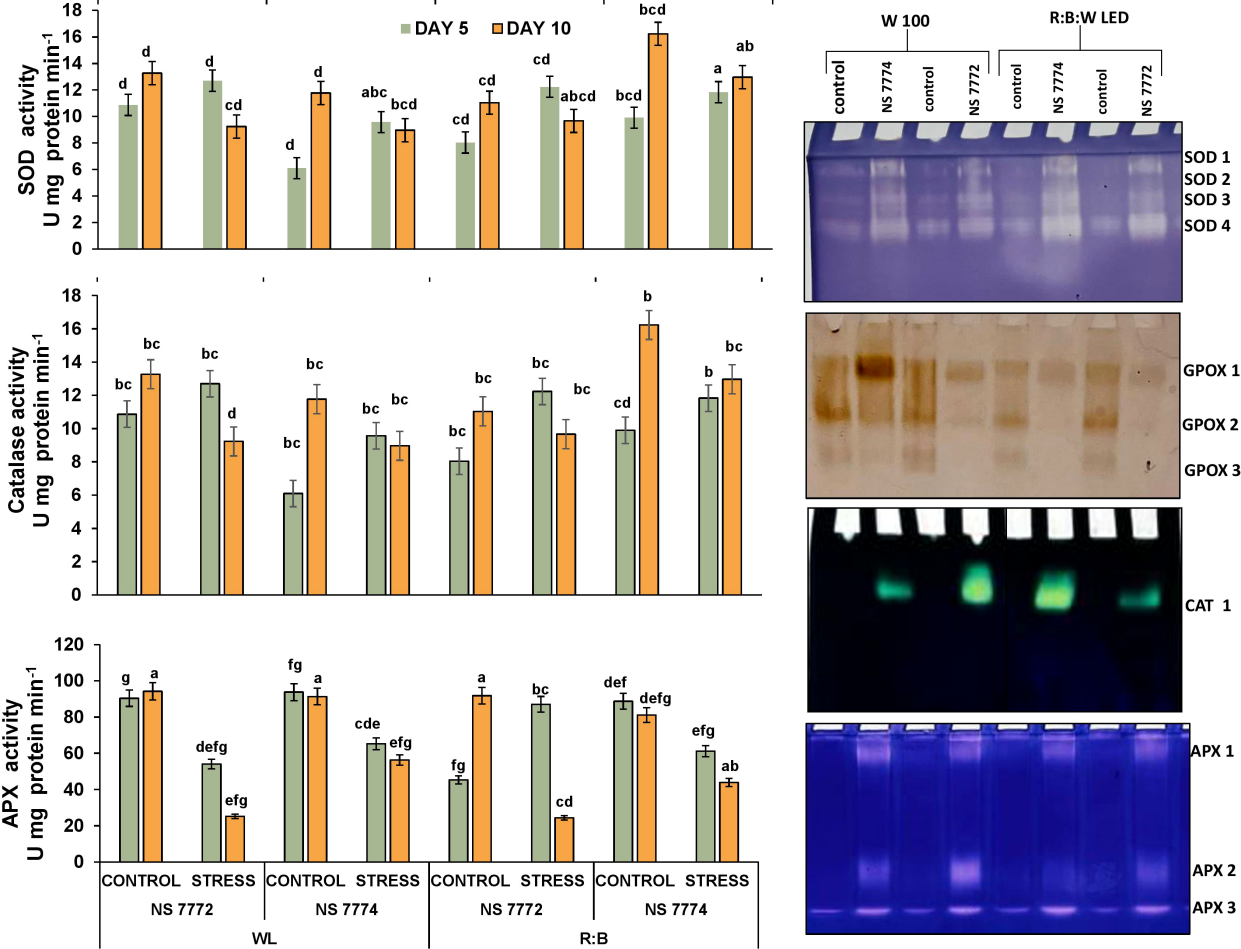
Visualisation of alterations in enzymatic antioxidant defenses and isoenzyme distribution. Antioxidant enzyme activity modifications (SOD, CAT, and APX) and Native PAGE based isozyme profiling of antioxidants (SOD, GPOX, CAT and APX) of okra genotypes (viz., NS 7772 and NS 7774, along with respective controls) under white fluorescent light system (WL) and multi-spectrum LED (R: B) light system during severe (day 10) drought stress at controlled micro-climatic conditions. Vertical bars indicate mean ± SE for n = 4. Means denoted by the different letters are significantly different at p ≤ 0.05 according to Tukey’s studentized range test.

The three key antioxidant enzymes superoxide dismutase (SOD), catalase (CAT), and ascorbate peroxidase (APX) was significantly altered by quantitative examination of antioxidant enzyme activities under drought stress on days 5 and 10 (**Figure 4A**). Under R: B LED light and WL (white fluorescent light), both NS 7772 and NS 7774. Under drought, SOD activity increased in both genotypes. But under R:B LEDs, NS 7774 showed the highest SOD activity, suggesting a better capacity to convert superoxide radicals (O_2_^-^) into hydrogen peroxide. NS 7772, on the other hand, showed the lowest SOD induction under WL, suggesting a poorer primary antioxidant response. Under LED light, catalase, which detoxifies hydrogen peroxide, rose in NS 7774 but stayed lower in NS 7772 under both light treatments, suggesting ineffective H2O_2_ detoxification. Under R: B, APX activity was also significantly elevated in NS 7774, suggesting its active participation in the ascorbate-glutathione cycle and precise control of H2O_2_. APX activity in NS 7772 rose during dryness but stayed low under both lighting conditions, suggesting a weaker redox balancing mechanism. These enzyme trends demonstrate that R: B LED light improves enzymatic antioxidant defense, especially in NS 7774, and are consistent with the reported reduction in ROS accumulation (**Figure 4**).

### 3.6 Isoenzyme profiling

Native PAGE investigation (**Figure 4**) demonstrated qualitative and intensity-dependent variation in isoenzymes for SOD, GPOX (guaiacol peroxidase), CAT, and APX across genotypes and treatments. Multiple SOD isoforms were found, among which SOD1 and SOD2 were more active in NS 7774 under LED exposure, indicating isoform-specific overexpression. Under WL, NS 7772 exhibited fainter bands, particularly for SOD 3 and 4. GPOX isozymes, which detoxify H_2_O_2_ through peroxidation, showed elevated band intensity in NS 7774, particularly under R:B: W LEDs, with GPOX 2 being the most prominent. These bands were reduced in NS 7772, especially at WL. CAT isoform (**Figure 4**) was also detected in all samples; however, NS 7774 showed higher expression under LED, which corresponded to the quantitative CAT activity. The band was faint or difficult to discern in NS 7772, strained with WL. APX 2 and APX 3 were more evident in NS 7774 under LED, indicating increased redox cycling. In NS 7772, band intensity and diversity were decreased, indicating that isoform activation was limited under stress.

### 3.7 Anatomical modifications and cellular adaptations

**Figure 5:**
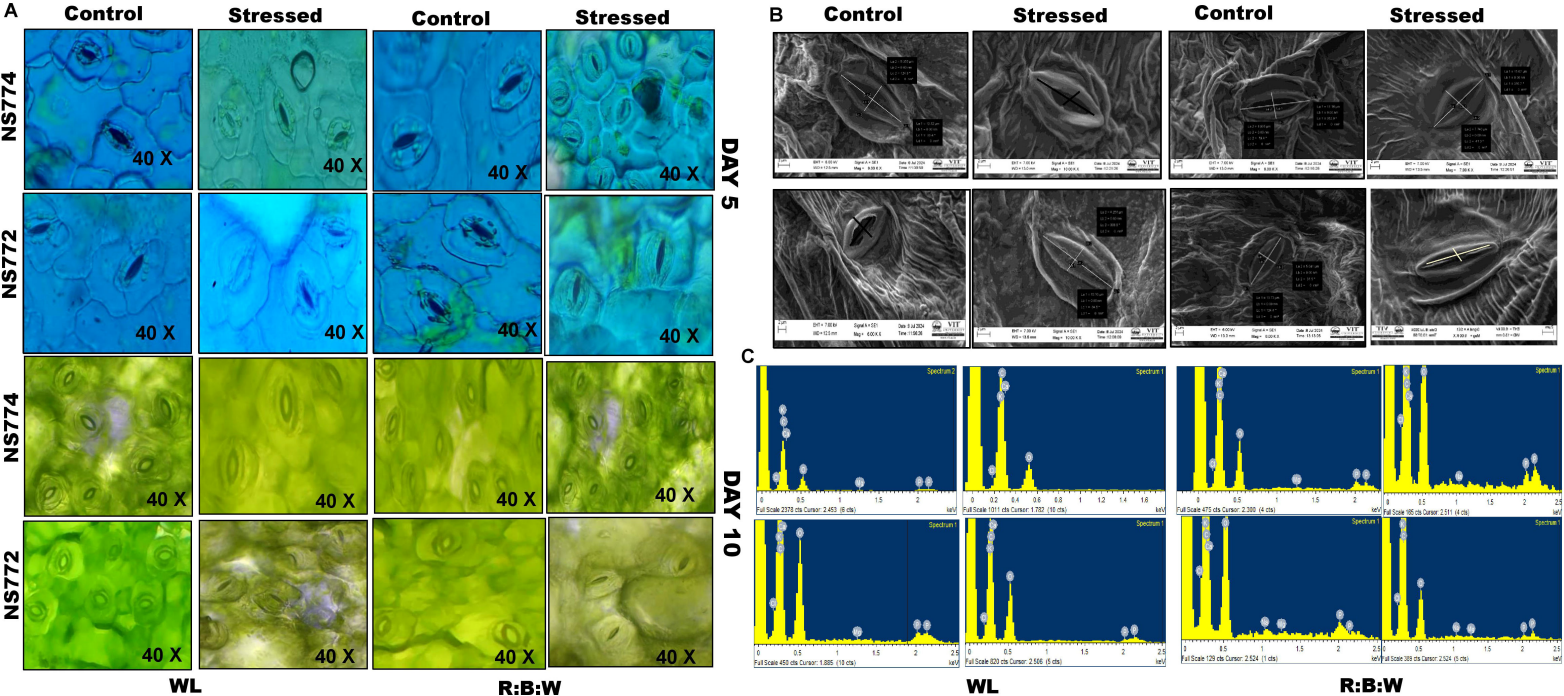
Morphological representation of **(A)** dark field and phase contrast microscopic images of stomata structure during mild (day 5) and severe (day 10) drought stress conditions. And **(B)** scanning electron microscopic images with **(C)** EDAX of okra genotypes (viz., NS 7772 and NS 7774) under white fluorescent light system (WL) and multi-spectrum LED (R:B) light system during severe (day 10) drought stress at controlled micro-climatic conditions.

On Day 5 (moderate drought) and Day 10 (severe drought), stomatal aperture response in NS 7772 and NS 7774 was assessed using dark-field and phase-contrast microscopy with WL and R:B LED treatments, respectively (**Figure 5**). Under mild drought conditions, NS 7774 under R: B had partially open stomata (**Figure 5A**) indicating a balanced regulation of gas exchange and water conservation. NS 7772 under WL showed early stress perception and impaired stomatal function due to premature stomatal closure or distortion. On Day 10, NS 7774 displayed more intact architecture in both lighting systems, while control plants maintained their morphology. On day 10, however, NS 7772 under WL displayed distorted, closed stomatal morphology and collapsed guard cells, suggesting irreversible stress effects and structural disintegration. Nonetheless, NS 7774 retained clear elliptical open stomata under LED, suggesting regulated water loss and continuous metabolic activity. These findings demonstrate that NS 7774 has improved stomatal flexibility and functional integrity, especially under R: B LED, which helps with drought adaptation. On Day 10, a scanning electron microscope was used to take surface architectural images (**Figure 4**), which showed the pore structure, stomatal density, and epidermal texture under severe drought circumstances. Significant drought-induced damage was shown by the sunken, disorganized stomata, shredded cuticle, and uneven surface textures that NS 7772 displayed during WL stress. NS 7774, on the other hand, showed better cell wall integrity and resistance to cuticular disruption under R: B LED stress by maintaining intact stomatal outlines, consistent pore spacing, and a smoother cuticular surface. Even under stress, LED treatments produced more distinct epidermal features in both genotypes than WL, with NS 7774 showing the most consistent morphology. Elemental alterations specific to the genotype were identified by EDAX analysis of leaf tissues on Day 10 under drought stress and different light treatments (**Figure 5c**). Carbon (C), oxygen (O), magnesium (Mg), sodium (Na), phosphorus (P), potassium (K), and calcium (Ca) were the elements discovered. K and Ca, which are essential for stomatal function, osmotic adjustment, and membrane stability, had smaller peaks in NS 7772 during the WL drought. This demonstrates ion leakage when under stress. On the other hand, following R: B LED therapy, NS 7774 retained greater Ca and K levels, suggesting enhanced ionic homeostasis and drought tolerance and P, which are necessary for energy metabolism and chlorophyll construction, were significantly more prevalent in NS 7774, demonstrating that metabolic activity was preserved. A popular stress indicator ion, sodium (Na), accumulated very little in both genotypes but much less in NS 7774, suggesting more effective ion exclusion mechanisms. These compositional patterns show that NS 7774 has better ionic stability and nutrient retention, especially under R: B LED lighting, which is correlated with better anatomical and physiological responses to drought.

### 3.8 Photosynthetic performance and chlorophyll fluorescence alterations

**Figure 6:**
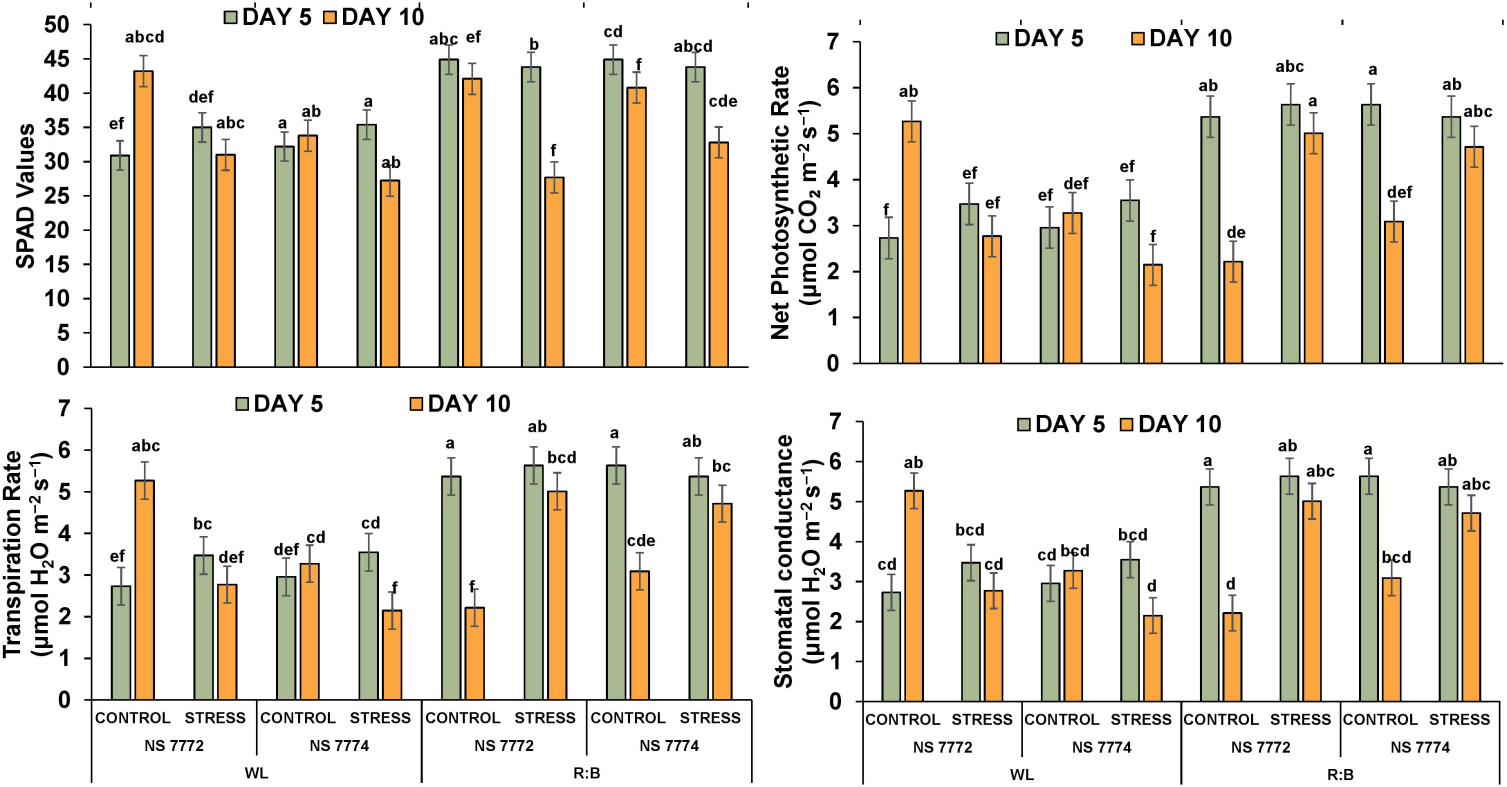
Impact of drought stress on photosynthetic efficiency indicators. Relative chlorophyll content, net photosynthetic rate, transpiration rate and stomatal conductance of okra genotypes (viz., NS 7772 and NS 7774, along with respective controls) under white fluorescent light system (WL) and multi-spectrum LED (R:B) light system during severe (day 10) drought stress at controlled micro-climatic conditions. Vertical bars indicate mean ± SE for n = 4 Means denoted by different letters are significantly different at p ≤ 0.05 according to the Tukey’s studentized range test.

Under drought stress, all genotypes and light treatments showed decreased chlorophyll concentration. While NS 7774 maintained noticeably higher SPAD values (**Figure 6**), especially under R: B light, indicating delayed chlorophyll breakdown, NS 7772 showed the greatest drop under WL stress on Day 10. In both genotypes, R: B light supported higher SPAD values than WL on both days, suggesting enhanced photoprotection and chloroplast stability. The NS 7774 R:B stress group had the highest significant SPAD retention, suggesting greater tolerance. In both WL and R: B, NS 7774 showed a higher photosynthetic rate than NS 7772 on Day 5, although the decline by Day 10 was more noticeable in WL. R: B stressed NS 7774 exhibited the least amount of photosynthesis loss, suggesting higher efficient carbon absorption and electron transfer. Significantly reduced rates were shown by NS 7772, especially under WL, suggesting subpar recovery mechanisms. Under WL control, NS 7772 showed more transpiration loss; however, under stress, especially on Day 10, this substantially decreased. Even under stress, NS 7774 maintained more consistent transpiration under R: B, probably as a result of better stomatal regulation. On Day 10, R: B treated NS 7774 exhibited the highest Transpiration, indicating its capacity to sustain gas exchange. Stomatal conductance followed a similar trajectory to transpiration. Both genotypes declined under stress, although R: B illumination reduced the decrease, particularly in NS 7774. On Day 10, conductance values in the NS 7774 R: B stress group were considerably higher than in the WL group, indicating improved functional stomatal responses and water-use efficiency under drought. Under WL stress, NS 7772 had the lowest stomatal conductance.

### 3.9 Differential proteomic expression profiling

**Figure 7:**
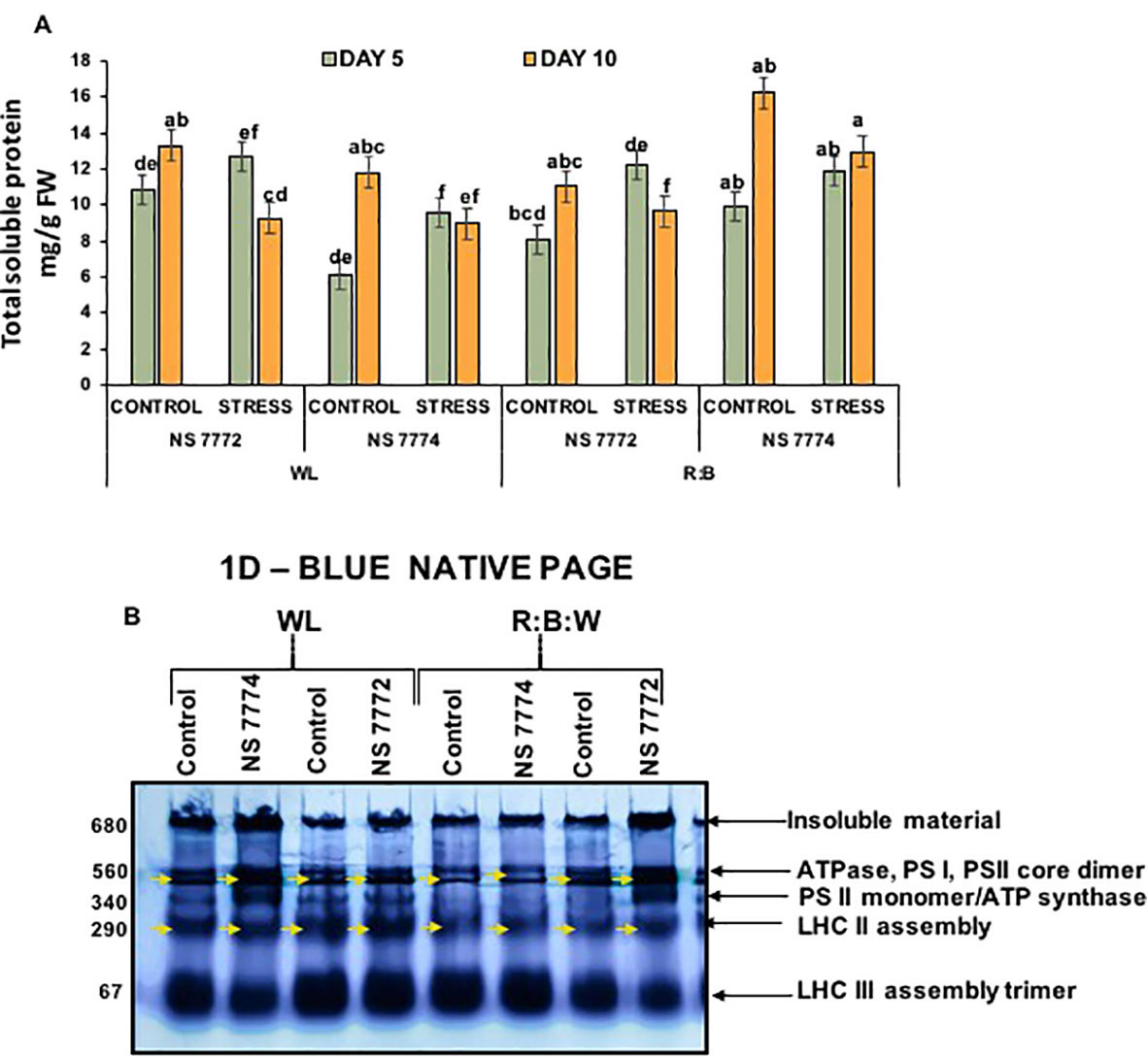
Stress-induced comparative proteomic analysis **(A)** Total soluble protein; Vertical bars indicate mean ± SE for n = 4. Means denoted by the different letters are significantly different at p ≤ 0.05 according to Tukey’s studentized range test **(B)**. First-dimension blue-native PAGE (BN-PAGE). Annotated bands reveals stress-responsive expression of okra genotypes (viz., NS 7772 and NS 7774, along with respective controls) under a white fluorescent light system (WL) and a multi-spectrum LED (R:B) light system during day 10 drought stress.

Total soluble protein content on day 10, NS 7772 under WL and NS 7774 under R: B stress showed a substantial increase in protein content, indicating that stress-protective proteins were synthesized more efficiently, refer **Figure 7**. Notably, R: B stressed NS 7774 retained the maximum protein content, whereas NS 7772 WL stress showed the lowest, indicating that light spectrum modulation effects protein accumulation under drought. Protein levels were usually greater on Day 10 than on Day 5, indicating a time-dependent adaptive response. Under drought stress, a particular protein alteration occurs in every genotype of okra.

The blue-native PAGE (BN-PAGE) approach permitted the resolution of intact thylakoid membrane protein complexes to investigate the stability and integrity of photosynthetic machinery in response to drought and light spectral treatments. Major complexes, including ATPase, PSI, PSII core dimers, PSII monomer/ATP synthase, LHC II assembly, and LHC II trimer, were identified by different bands on the resolved gel. A considerable decrease in band intensity was seen under white light (WL) stress, specifically in NS 7772, especially in the PSII dimer and LHC II areas. This suggests that the light-harvesting and core complexes of photosystem II have undergone significant disassembly or degradation. Poor thylakoid organization and oxidative damage brought on by drought are probably the causes of this disassembly, which lowers photosynthetic effectiveness. Conversely, red, blue, and white (R:B) Plants treated with LEDs, particularly NS 7774, showed more distinct and stronger bands across the profile. This suggests that LED spectrum tuning promotes proteome stability, especially of crucial photosynthetic structures, as it shows the retention of native protein complexes even under drought stress. This implies that the R:B spectrum supports an active stress-adaptive proteome remodeling. These new patches could be drought-inducible signaling molecules, antioxidant enzymes (like APX and SOD), or heat shock proteins (HSPs), which maintain cellular homeostasis under stress.

### 3.10 Multivariate analysis of physiological and proteomic interactions

**Figure 8:**
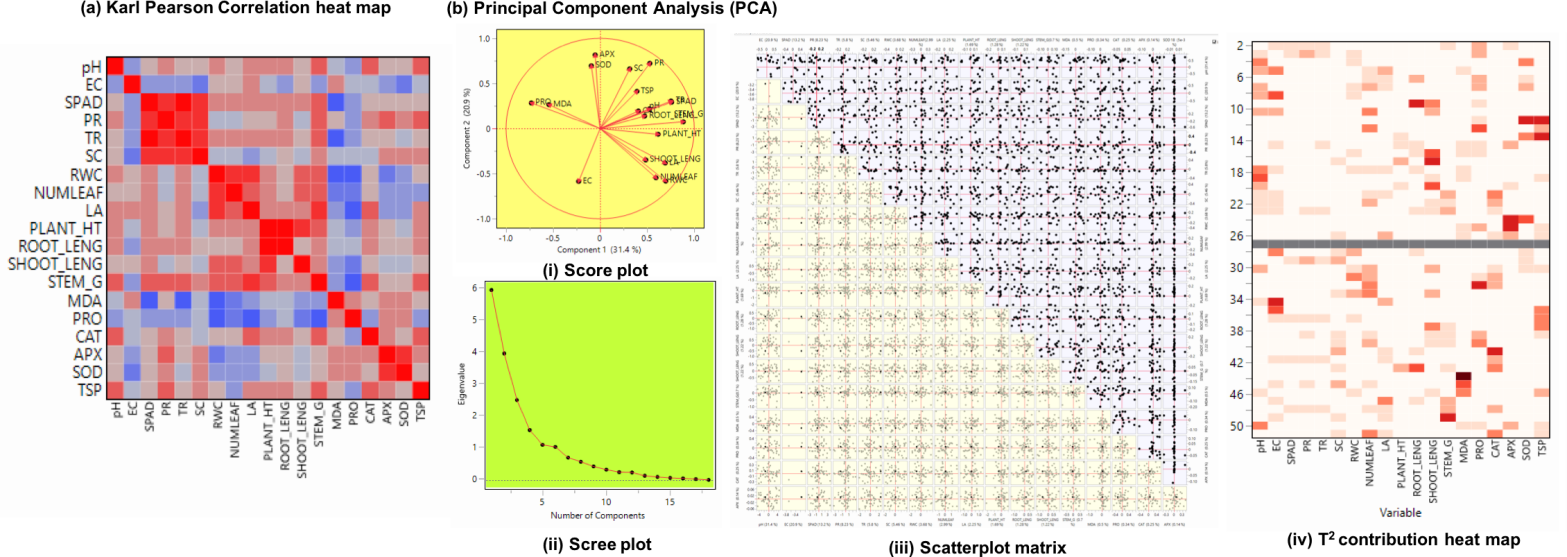
**(A)** Karl Pearson’s correlation (r) heat map of physiological and biochemical traits followed by **(B)** Principle component analysis – **(i)** Score plot visualizing clustering based on trait performance **(ii)** Scree plot showing proportion of variance explained by each principal component **(iii)** Scatterplot matrix depicting influence of each component on principal component (iv) T^2^ contribution heat map identifies variables contributing significantly to sample variability of okra genotypes (viz., NS 7772 and NS 7774, along with respective controls) under white fluorescent light system (WL) and a multi-spectrum LED (R:B) light system during day 10 drought stress.

To evaluate the associations and key contributing factors influencing drought tolerance in okra genotypes Karl Pearson’s correlation and Principle component analysis was performed (**Figure 8**). Important morpho-physiological characteristics, such as root length, shoot length, plant height, stem girth, and relative water content (RWC), showed strong positive associations, suggesting that they coordinate to sustain growth during drought. Positive correlations between photosynthetic pigments and antioxidant enzymes SPAD, CAT, APX, and SOD indicate that mechanisms for mitigating oxidative stress are tightly controlled. MDA’s role as a biomarker of stress-induced cellular damage was confirmed by its negative associations with physiological traits. Surprisingly, proline (PRO) and total soluble protein (TSP) demonstrated a substantial relation with antioxidative parameters, supporting their role in osmotic adjustment and stress adaptation.

Sample adequacy was confirmed, and all variables were standardized using z-score normalization before PCA analysis. Loadings ≥ 0.50 were considered significant for component interpretation because of the biological interdependence among the variables. With high loading of photosynthetic rate, transpiration rate, stomatal conductance, RWC, shoot length, and antioxidants (SOD and CAT), the PC1 demonstrated significant drought resistance and contributed significantly to the total variation of roughly 31.36%. The negative clustering of MDA, SOD, APX and PR (peroxidase) along PC2 (20.88%) suggests that drought tolerance is inversely correlated with their overexpression. Differential trait groups were shown by the biplot’s vector length and angular spacing between variables. The scatterplot matrix depicted trait association across all treatments and genotypes. There were other clusters of linear relationships found, especially between CAT and SOD and between RWC and growth characteristics, indicating that these traits are associated in mitigating drought. Under R: B stress, NS 7774 clearly indicated a high level of adaptive trait expression. On the other hand, NS 7772 exhibited weak drought adaptation under white light stress. The relative contribution of each trait to the total variability of the sample was shown visually via Hotelling’s T2 contribution heatma Interestingly, MDA, EC, and PR significantly increased the variability in NS 7772 treated with WL, highlighting oxidative damage in the presence of WL light. In contrast, the biggest contributors in R: B-treated NS 7774 were SPAD, RWC, root length, shoot length, CAT, and APX, contributing towards stress tolerance.

## 4 Discussion

Through integrated photoreceptor, hormonal, and metabolic processes, certain light spectra influence drought tolerance. Drought alleviation is mostly driven by blue light. According to recent research, antioxidant enzymes, including SOD, CAT, and APX, are quickly elevated by cryptochrome and phototropin-mediated signaling, which lowers ROS accumulation under water deprivation (Vicente-Serrano et al., 2022; Chibani et al., 2025). The blue light-induced increase in ABA sensitivity in guard cells, which enhances stomatal conductivity during early drought progression, is complemented by increased ROS scavenging according to Li et al., 2024. When stomatal conductance is decreased, these reactions work together to preserve membrane stability and photochemical efficiency. Through phytochrome signaling, red light plays a major role in drought adaptation. The thorough physiological, photosynthetic, and proteomic analyses show that the okra genotypes respond differently to drought under different light spectra, white light (WL), and R:B lights. These findings have important ramifications for rootstock selection during drought stress (Shahzad et al., 2023; Collado et al., 2024; Alrajhi et al., 2023). NS 7774 sustained lower leaf temperatures under R: B, according to canopy temperature imaging, indicating more effective stomatal control and transpiration cooling processes essential for photoprotection under water-deficit circumstances (Brestic et al., 2018). These characteristics improve drought adaptation by facilitating gas exchange and water intake even in conditions of limited supply (Razi and Muneer, 2023). These findings are consistent with those of Kumar et al. (2019); Eltigani et al. (2021), and Kumar et al. (2025).

Under drought, ABA accumulates rapidly, increasing stomatal closure to decrease water loss and activating transcriptional upregulation of antioxidant enzymes (APX, CAT, SOD) and osmoprotectants such as proline. Higher proline concentration, which supports osmotic equilibrium, and improved antioxidant defenses, which reduce membrane lipid peroxidation and prevent excessive MDA generation, which are linked to increased ABA. ABA interacts with both hormones. Melatonin (MT) and jasmonic acid (JA) can increase proline synthesis and antioxidant enzyme activity, which can reduce MDA and improve membrane integrity. MT complements the actions of ABA by increasing the membrane stability index (MSI), APX, and CAT activities. Biochemical reactions revealed that under R: B, MDA levels were lower and chlorophyll retention (SPAD values) was higher, indicating a decrease in oxidative stress (Ahmad et al., 2020; Kang et al., 2022). It’s interesting to note that while both genotypes accumulated proline, a compatible osmolyte, NS7774 showed a longer-lasting accumulation under R: B at both time periods. This is consistent with previous findings in Solanaceae and Brassicaceae under spectrum manipulation, indicating improved osmotic adjustment and ROS buffering (Razi et al., 2024; Vajjiravel et al., 2024; Xu et al., 2022). Beyond its Osmo protective role, proline works in concert with enzymatic antioxidants to stabilize proteins and scavenge reactive oxygen species (ROS). Hormonal signaling increases its production. NS 7774 typically accumulate more proline, which is positively correlated with improved stress tolerance (Zaidi et al., 2022; Gul et al., 2025).

To protect cells from oxidative damage caused by reactive oxygen species (ROS), the plant defense system mostly depends on antioxidant enzymes. Stressors increase the production of ROS, including superoxide anion (O_2_^-^), hydrogen peroxide (H2O_2_), and hydroxyl radicals (OH-). Proteins, lipids, and nucleic acids may be harmed by excessive ROS generation, which may ultimately lead to cell death (Gill and Tuteja, 2010). SOD, CAT, and APX are some of the primary enzymatic antioxidants. Superoxide dismutase (SOD) converts superoxide radicals (O_2_^-^) into hydrogen peroxide (H2O_2_) (Mittler, 2002). Catalase (CAT) converts hydrogen peroxide into water and oxygen (Apel and Hirt, 2004). Ascorbate peroxidase (APX) converts H_2_O_2_ to water by using ascorbate as an electron donor (Foyer and Noctor, 2005). The pool of reduced glutathione, which is essential for scavenging ROS through the ascorbate-glutathione cycle, is maintained by glutathione reductase (GR). Hence, it was also essential to study the superoxide radicals (O_2_^-^) and hydrogen peroxide (H_2_O_2_) deposition levels (Kumar et al., 2019; Farooq et al., 2009). In this study, on day 5 and day 10, the NS 7774 genotype under R: B LED treatment showed the highest expression, affirming the role of reactive oxygen species detoxification in drought resilience (Shibaeva et al., 2022). The antioxidant expression levels are in line with the isoenzyme patterns of the respective enzymesIsoform-specific profiles revealed genotype-specific control of antioxidant isozymes; in plants treated with WL, NS 7774 under R:B expressed distinct bands not present in NS 7772. This demonstrates the proteome flexibility that could support enhanced cellular defense systems (Abbas et al., 2023). The band diversity and qualitative strength of isoenzymes support proteome-level plasticity in NS 7774 in response to light and drought stimuli. H_2_O_2_ and O_2_^-^ radicals deposited under drought-stressed and control samples are consistent with the antioxidant expression pattern; NS 7774 under R: B outperforms, followed by NS 7774 under WL, demonstrating resistance on both days 5 and 10. The intake of critical macro elements (Ca, Mg, K, and P) was confirmed by elemental analysis (EDAX), with NS 7774 exhibiting marginally superior nutrient retention under stress in R:B, followed by WL, indicating stabilized cell wall and membranes, better osmotic adjustment, sustained photosynthesis and stomatal control, and improved root proliferation (Sakuranaka et al., 2022; Hasanuzzaman et al., 2019; Chaves et al., 2009). Although drought universally reduced shoot and root length, leaf area, and stem girth, the decline was minimal under R: B, particularly for NS 7774, indicating improved morphological plasticity. Root system resilience is often associated with better soil exploration and water foraging under deficit stress (Kong et al., 2023; Formisano et al., 2022; Hernandez et al., 2016), further validating NS 7774 under both R: B followed by WL marked the highest root length. The fate of protein complexes under drought is greatly influenced by the quality of the light, and the R: B spectrum improves proteostasis by modifying light-signal transduction pathways (such as cryptochromes and phytochromes) that control chloroplast function (Zhu et al., 2024; Wang et al., 2021). In R: B-exposed NS 7774 plants, the combination of stable native complexes (BN-PAGE) exhibits a light-enhanced adaptive proteome response, indicating increased synthesis of stress-adaptive proteins, which thereby improves water retention, improves nitrogen metabolism, better ROS protection, and reduces cell shrinkage. Total soluble protein (TSP) content remained highest in NS 7774 under R: B, aligning with enhanced maintenance of photosynthetic machinery. TSP improved water retention and osmo protection by activating molecular defenses against drought. BN−PAGE results supported this, revealing stable complexes of PSII, PSI, and LHC even under stress in NS 7774 under R: B followed by WL. NS 7774 in R: B, likely representing LEA proteins, chaperonins, and stress-induced proteases involved in cellular homeostasis (Mirzahosseini et al., 2020; Kosova et al., 2011). Multivariate statistical analysis consolidated these insights. Pearson’s heatmap highlighted strong positive correlations among RWC, chlorophyll, protein, antioxidant enzymes, and morphological parameters, while MDA correlated negatively (Malekzadeh et al., 2024). Six major components (eigenvalue >1) were identified by principal component analysis, and taken together, they cumulatively accounted for 84.94% of the variance. High positive loadings for photosynthetic rate, transpiration rate, stomatal conductance, RWC, leaf area, shoot length, proline, SOD, and CAT were observed in PC1, which explained 31.36% of the variance, whose elements are associated with the primary physiological drought-tolerance axis, combining antioxidant metabolism, water retention, and gas-exchange efficiency. EC and APX activity were linked to PC2 (20.88%), suggesting antioxidant detoxification and membrane stability. Strong contributions from MDA, proline, and SOD were seen in PC3 (13.20%), which represents compensatory osmolyte-antioxidant responses and oxidative damage. PC5 represented root length and antioxidant enzymes, while PC4 recorded structural characteristics such as plant height, leaf count, and stem circumference. PC6 reported a little amount of residual variation.

Overall, drought-resilient lines scored higher in PC1 and PC2, indicating that the main factors influencing drought tolerance are greater gas exchange, water conservation, and antioxidant potential. Hotelling’s T² plot identified chlorophyll, protein content, shoot length, and APX as the primary discriminatory variables, affirming their utility in genotype screening under drought. NS 7774 shows a superior drought resilience profile under both R: B and WL, with improved performance in R: B LED light followed by WL, according to the integration of physiological, biochemical, and proteomic indices. Stable water relations, decreased lipid peroxidation, robust antioxidant defenses, maintained photosynthetic protein complexes, and root-shoot integrity all contribute to this resilience. The overall data indicates that NS 7774 under R: B LED showed the most comprehensive improvement, even if NS 7772 showed positive responses in individual feature. Red:blue:white (R:B) spectral combinations, in particular, have shown promise in enhancing drought resilience through the optimization of photosynthetic activity and the activation of protective signaling networks. This highlights the potential of light spectrum modulation as a targeted strategy to enhance crop tolerance under climate-induced stress and validates NS 7774 as a promising rootstock genotype for drought-prone environments.

## 5 Conclusions

Light spectrum application and modifications can significantly affect the morphology, biochemical enzymes, and physiology by modulating the multiprotein complex of thylakoids in the chloroplast. This study highlighted the superior drought tolerance of the okra genotype NS 7774 compared to NS 7772 under contrasting light spectra, with red: blue (R: B) LED showing the most beneficial effects thereby establishing NS 7774 as a promising genotype for rootstock selection and emphasized the potential of light spectrum R: B spectral lights as a strategic tool to enhance crop resilience under drought stress. Essentially, the research proves that light spectrum quality affects defense mechanisms and stress signaling in addition to energy, which enables drought-resistant okra genotypes like NS7774 to thrive in water-limited environments. Spectral optimization is a useful and scientific method for enhancing crop resilience in contemporary agriculture as the frequency of drought episodes rises. However, light spectral influence in graft healing, the mechanisms involved, and the extent of improvement of graft success are yet to be explored. Further future investigations are underpinned at the field level to validate the feasibility and suitability of the present study.

## Data Availability

The original contributions presented in the study are publicly available. This data can be found here: https://zenodo.org/records/17862994.
