# Supplementary material for: Spectral light quality regulates photosynthesis and thylakoidal protein complexes to improve drought tolerance in okra rootstocks
Source: Front Plant Sci. 2026 Jan 5;16:1706708. doi: 10.3389/fpls.2025.1706708 (PMC12813016; doi:10.3389/fpls.2025.1706708)
Supplement: Supplementary file 1 [file Presentation1.pptx]

## Slide 1
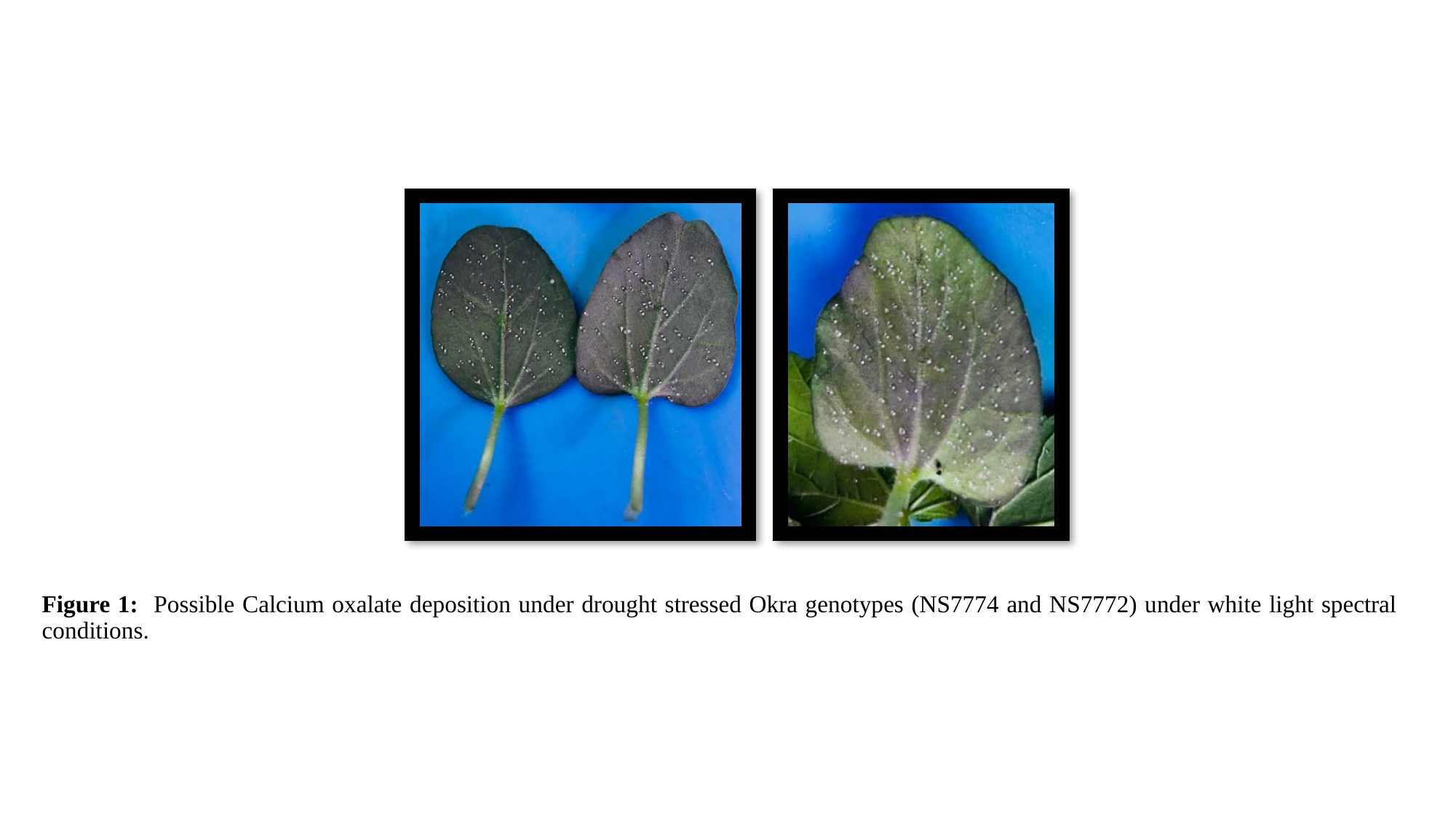

Figure 1: Possible Calcium oxalate deposition under drought stressed Okra genotypes (NS7774 and NS7772) under white light spectral conditions.

## Slide 2
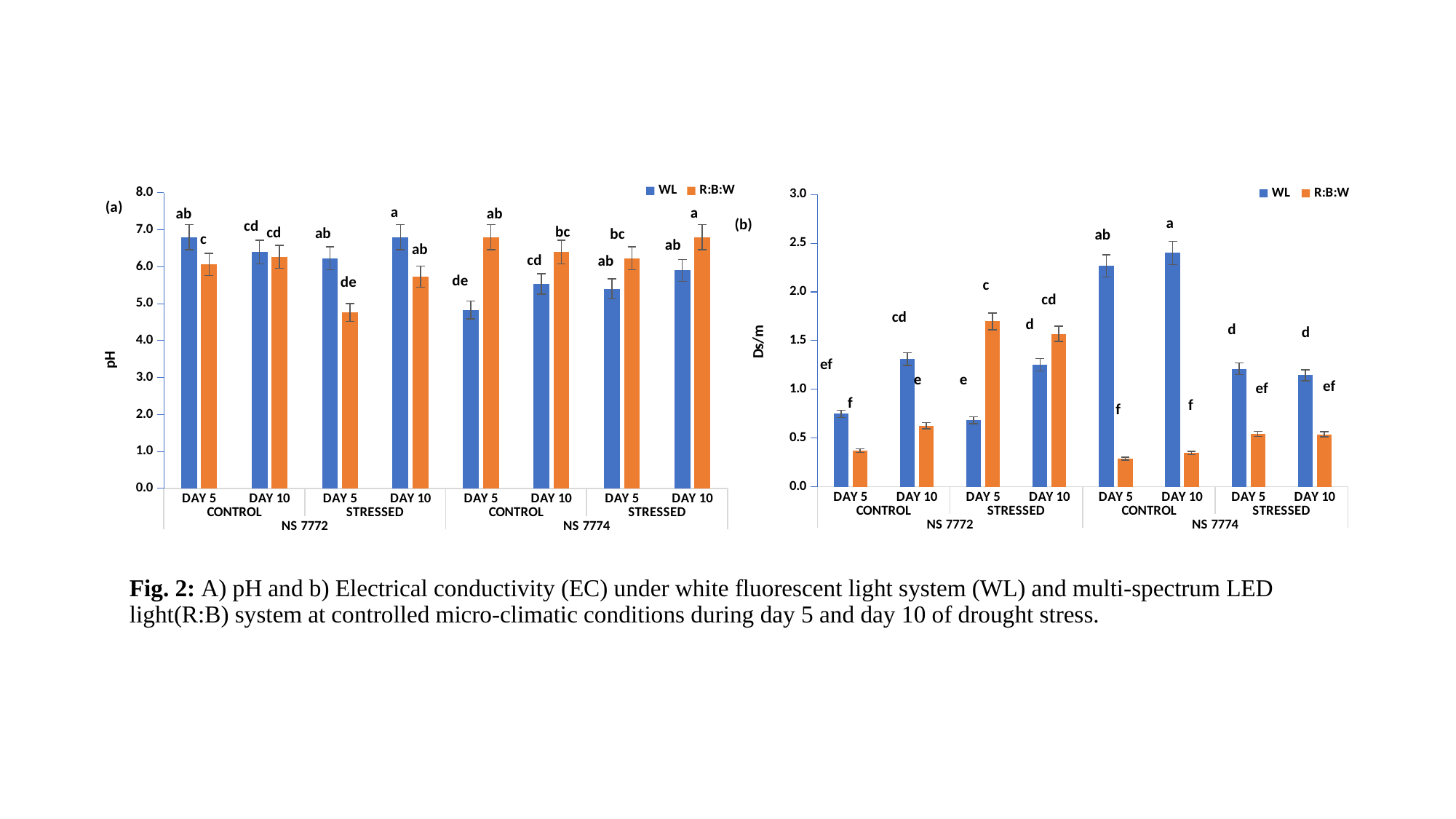

### Chart
| Category | WL | R:B:W |
|---|---|---|
| DAY 5 | 6.8 | 6.06 |
| DAY 10 | 6.4 | 6.267 |
| DAY 5 | 6.23 | 4.76 |
| DAY 10 | 6.8 | 5.73 |
| DAY 5 | 4.83 | 6.8 |
| DAY 10 | 5.53 | 6.4 |
| DAY 5 | 5.4 | 6.23 |
| DAY 10 | 5.9 | 6.8 |a
a
ab
ab
cd
bc
cd
ab
bc
c
ab
ab
cd
ab
de
de
### Chart
| Category | WL | R:B:W |
|---|---|---|
| DAY 5 | 0.748 | 0.37033333333333335 |
| DAY 10 | 1.311 | 0.625 |
| DAY 5 | 0.682 | 1.6973333333333331 |
| DAY 10 | 1.252 | 1.5693333333333335 |
| DAY 5 | 2.267 | 0.2866666666666667 |
| DAY 10 | 2.4 | 0.34600000000000003 |
| DAY 5 | 1.21 | 0.541 |
| DAY 10 | 1.144 | 0.538 |(a)
a
(b)
ab
c
cd
cd
d
d
d
ef
e
e
ef
ef
f
f
f
Fig. 2: A) pH and b) Electrical conductivity (EC) under white fluorescent light system (WL) and multi-spectrum LED light(R:B) system at controlled micro-climatic conditions during day 5 and day 10 of drought stress.

## Slide 3
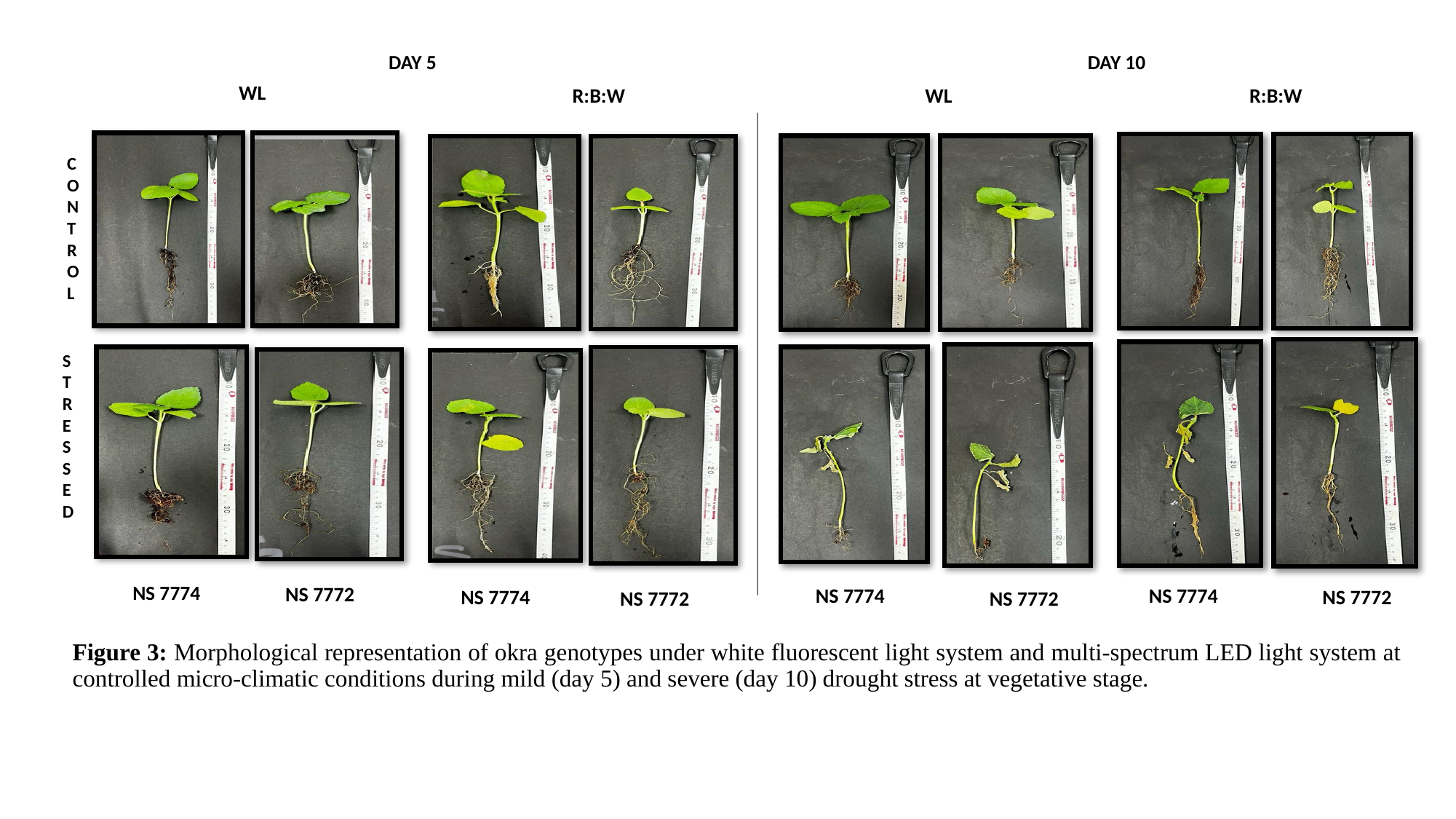

DAY 10
DAY 5
WL
R:B:W
R:B:W
WL
C
O
N
T
R
O
L
S
T
R
E
S
S
E
D
NS 7774
NS 7772
NS 7774
NS 7774
NS 7774
NS 7772
NS 7772
NS 7772
Figure 3: Morphological representation of okra genotypes under white fluorescent light system and multi-spectrum LED light system at controlled micro-climatic conditions during mild (day 5) and severe (day 10) drought stress at vegetative stage.

## Slide 4
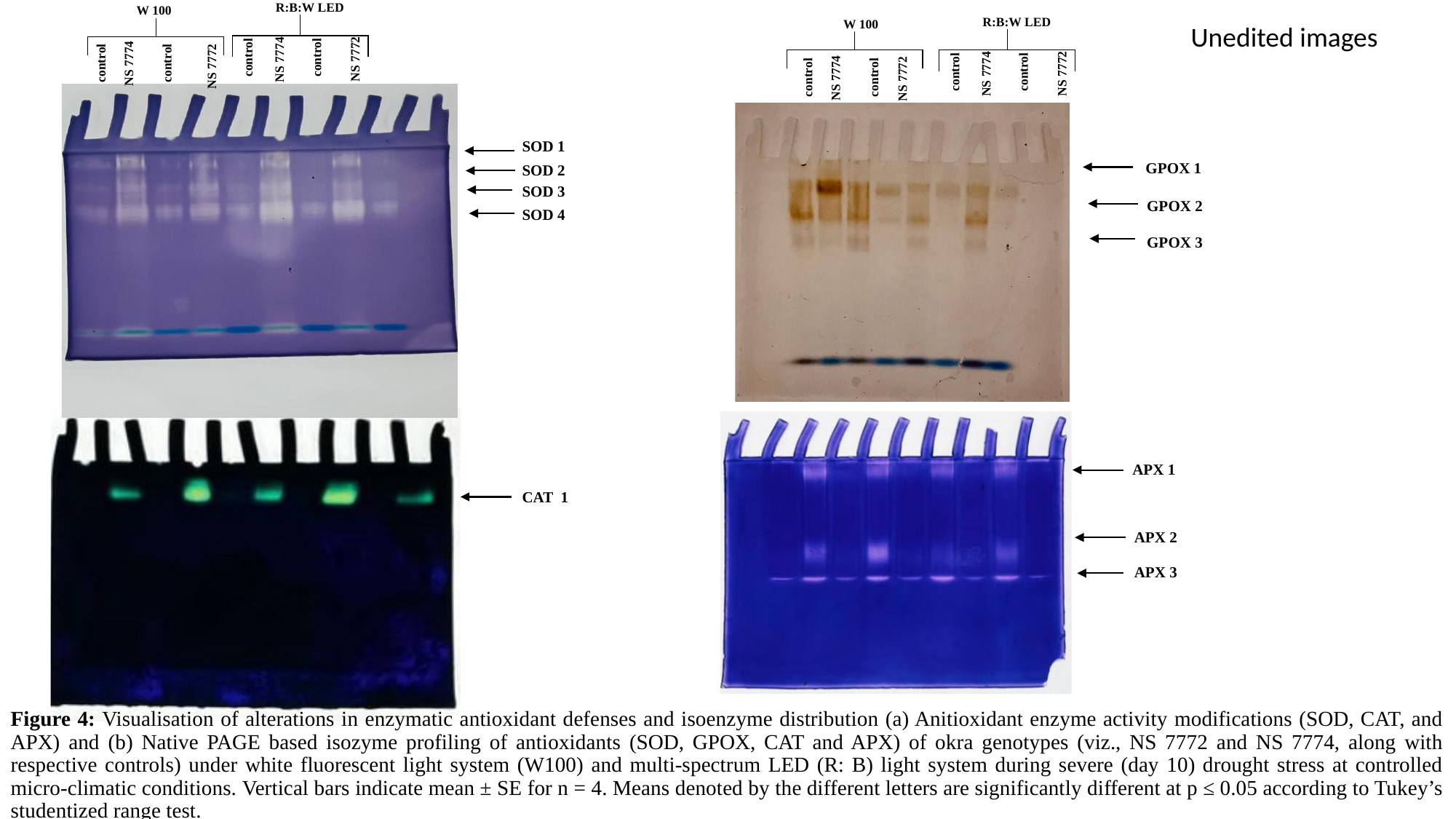

R:B:W LED
W 100
R:B:W LED
W 100
Unedited images
control
control
NS 7772
NS 7774
control
control
NS 7774
NS 7772
control
control
NS 7772
NS 7774
control
control
NS 7774
NS 7772
SOD 1
GPOX 1
SOD 2
SOD 3
GPOX 2
SOD 4
GPOX 3
APX 1
CAT 1
APX 2
APX 3
Figure 4: Visualisation of alterations in enzymatic antioxidant defenses and isoenzyme distribution (a) Anitioxidant enzyme activity modifications (SOD, CAT, and APX) and (b) Native PAGE based isozyme profiling of antioxidants (SOD, GPOX, CAT and APX) of okra genotypes (viz., NS 7772 and NS 7774, along with respective controls) under white fluorescent light system (W100) and multi-spectrum LED (R: B) light system during severe (day 10) drought stress at controlled micro-climatic conditions. Vertical bars indicate mean ± SE for n = 4. Means denoted by the different letters are significantly different at p ≤ 0.05 according to Tukey’s studentized range test.

## Slide 5
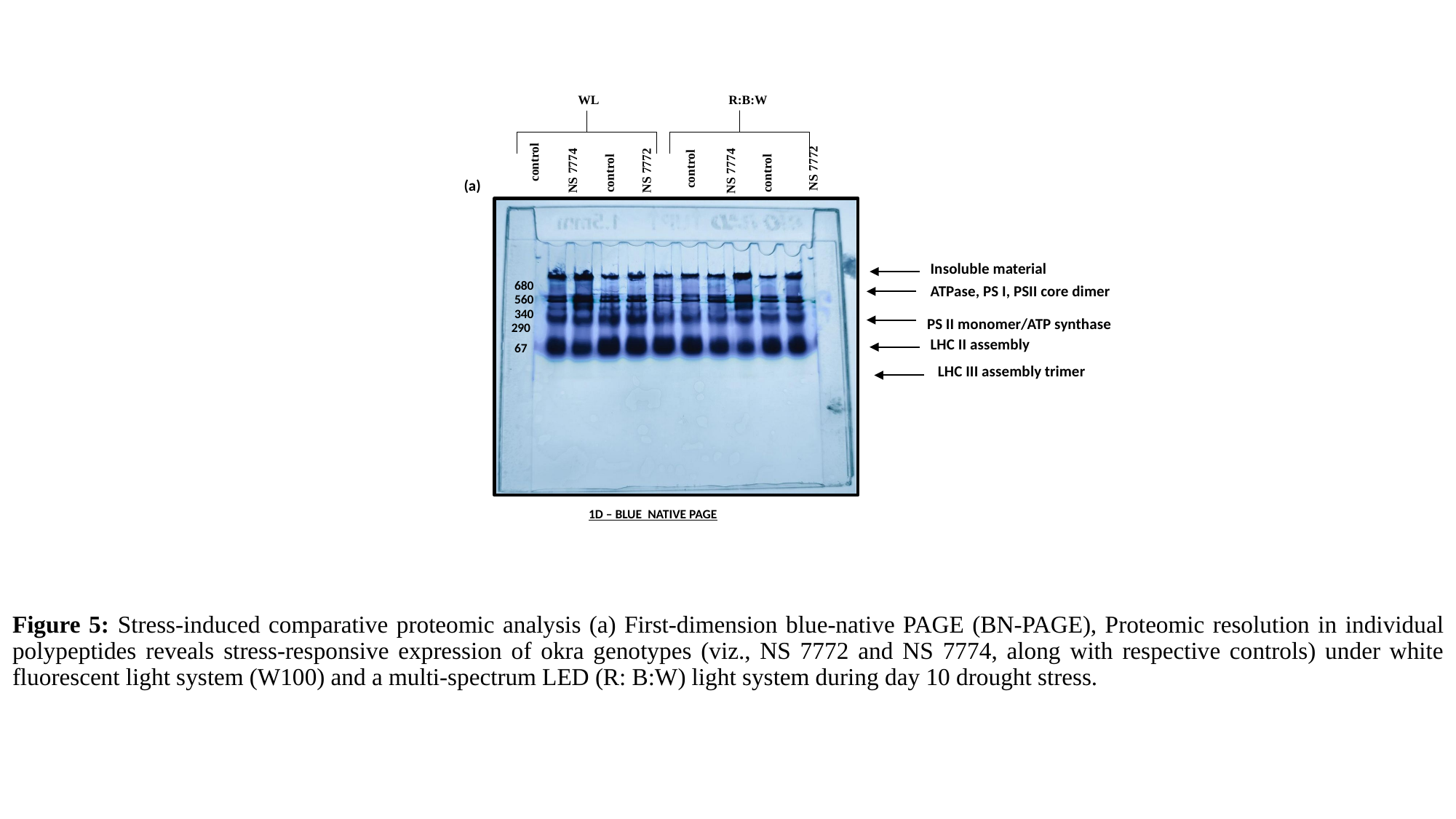

R:B:W
WL
control
control
NS 7772
control
control
NS 7772
NS 7774
NS 7774
(a)
Insoluble material
680
ATPase, PS I, PSII core dimer
560
340
PS II monomer/ATP synthase
290
LHC II assembly
67
LHC III assembly trimer
1D – BLUE NATIVE PAGE
Figure 5: Stress-induced comparative proteomic analysis (a) First-dimension blue-native PAGE (BN-PAGE), Proteomic resolution in individual polypeptides reveals stress-responsive expression of okra genotypes (viz., NS 7772 and NS 7774, along with respective controls) under white fluorescent light system (W100) and a multi-spectrum LED (R: B:W) light system during day 10 drought stress.

## Slide 6
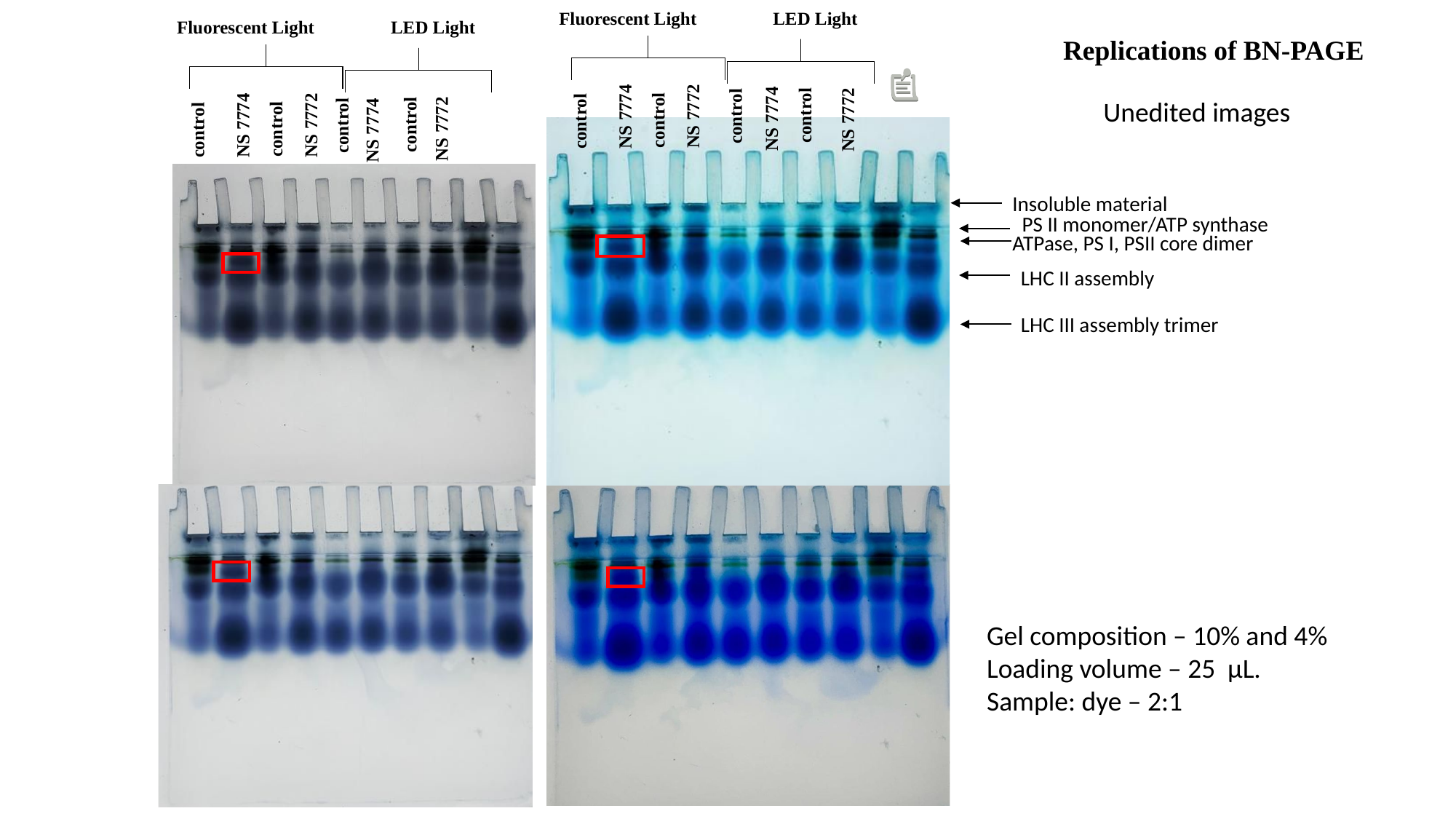

Fluorescent Light
LED Light
Fluorescent Light
LED Light
Replications of BN-PAGE
Unedited images
control
control
control
NS 7772
NS 7774
control
NS 7774
NS 7772
control
control
control
NS 7772
NS 7774
control
NS 7772
NS 7774
Insoluble material
PS II monomer/ATP synthase
ATPase, PS I, PSII core dimer
LHC II assembly
LHC III assembly trimer
Gel composition – 10% and 4%
Loading volume – 25 µL.
Sample: dye – 2:1
